# Extended automated quantification algorithm (AQuA) for targeted ^1^H NMR metabolomics of highly complex samples: application to plant root exudates

**DOI:** 10.1007/s11306-023-02073-z

**Published:** 2023-12-23

**Authors:** Elin Alexandersson, Corine Sandström, Johan Meijer, Gustav Nestor, Anders Broberg, Hanna E. Röhnisch

**Affiliations:** 1 Department of Molecular Sciences, Swedish University of Agricultural Sciences, Uppsala, Sweden; 2 Department of Plant Biology, Swedish University of Agricultural Sciences, Uppsala, Sweden

**Keywords:** Targeted metabolomics, Automated quantification, Baseline correction, AQuA, Root exudate, NMR

## Abstract

**Introduction:**

The Automated Quantification Algorithm (AQuA) is a rapid and efficient method for targeted NMR-based metabolomics, currently optimised for blood plasma. AQuA quantifies metabolites from 1D-^1^H NMR spectra based on the height of only one signal per metabolite, which minimises the computational time and workload of the method without compromising the quantification accuracy.

**Objectives:**

To develop a fast and computationally efficient extension of AQuA for quantification of selected metabolites in highly complex samples, with minimal prior sample preparation. In particular, the method should be capable of handling interferences caused by broad background signals.

**Methods:**

An automatic baseline correction function was combined with AQuA into an automated workflow, the extended AQuA, for quantification of metabolites in plant root exudate NMR spectra that contained broad background signals and baseline distortions. The approach was evaluated using simulations as well as a spike-in experiment in which known metabolite amounts were added to a complex sample matrix.

**Results:**

The extended AQuA enables accurate quantification of metabolites in 1D-^1^H NMR spectra with varying complexity. The method is very fast (< 1 s per spectrum) and can be fully automated.

**Conclusions:**

The extended AQuA is an automated quantification method intended for 1D-^1^H NMR spectra containing broad background signals and baseline distortions. Although the method was developed for plant root exudates, it should be readily applicable to any NMR spectra displaying similar issues as it is purely computational and applied to NMR spectra post-acquisition.

**Supplementary Information:**

The online version contains supplementary material available at 10.1007/s11306-023-02073-z.

## Introduction

Nuclear magnetic resonance (NMR) spectroscopy is commonly used in metabolomics for identification and quantification of metabolites in different biological samples (Crook & Powers, 2020). NMR has many advantages; it is inherently quantitative, highly reproducible, non-destructive, and enables analysis of compounds with different chemical properties in one single experiment. However, the complex mixtures of natural products that are studied in metabolomics typically yield complicated 1D-^1^H NMR spectra with extensive spectral overlap, which can make both identification and quantification of individual metabolites challenging. Spectral overlap occurs because one metabolite can generate several NMR signals, and signals from different compounds often appear at similar chemical shifts. The resulting signal interferences are especially problematic for quantitative studies because concentrations of individual metabolites will be overestimated unless the interferences are properly accounted for. Two-dimensional NMR experiments can be used to increase signal dispersion, but 2D spectra typically take longer time both to acquire and to analyse than 1D spectra. Furthermore, quantification based on 2D spectra is not straightforward since the intensity of individual peaks is influenced by their coupling constants and transverse relaxation times. Accordingly, calibration with pure reference compounds, either externally or internally, is required for accurate quantification (Crook & Powers, 2020; Martineau et al., 2020). Therefore, 1D-^1^H NMR experiments are still the most common in high-throughput studies and there continues to be a high demand for methods that can accurately quantify metabolites based on 1D-^1^H spectra. Various approaches have been developed, both manual (Weljie et al., 2006) and automated (Zheng et al., 2011; Hao et al., 2012; Ravanbakhsh et al., 2015; Tardivel et al., 2017; Lefort et al., 2019; Häckl et al., 2021; Rout et al., 2023).

An Automated Quantification Algorithm (AQuA) for targeted metabolomics has previously been developed in our group (Röhnisch et al., 2018, 2021). This method quantifies metabolites from 1D-^1^H NMR spectra using only one signal per metabolite, which reduces the computational time and workload substantially compared to e.g. curve-fitting quantification algorithms (Zheng et al., 2011; Hao et al., 2012; Ravanbakhsh et al., 2015; Tardivel et al., 2017). At the same time, AQuA corrects for signal interferences between different metabolites as well as inter-spectral variation in signal position. Currently, AQuA is optimised for ultra-filtered human plasma samples but it would be desirable to extend its use to other, more heterogeneous, sample types as well, preferably without any time-consuming sample preparation.

**Fig. 1:**
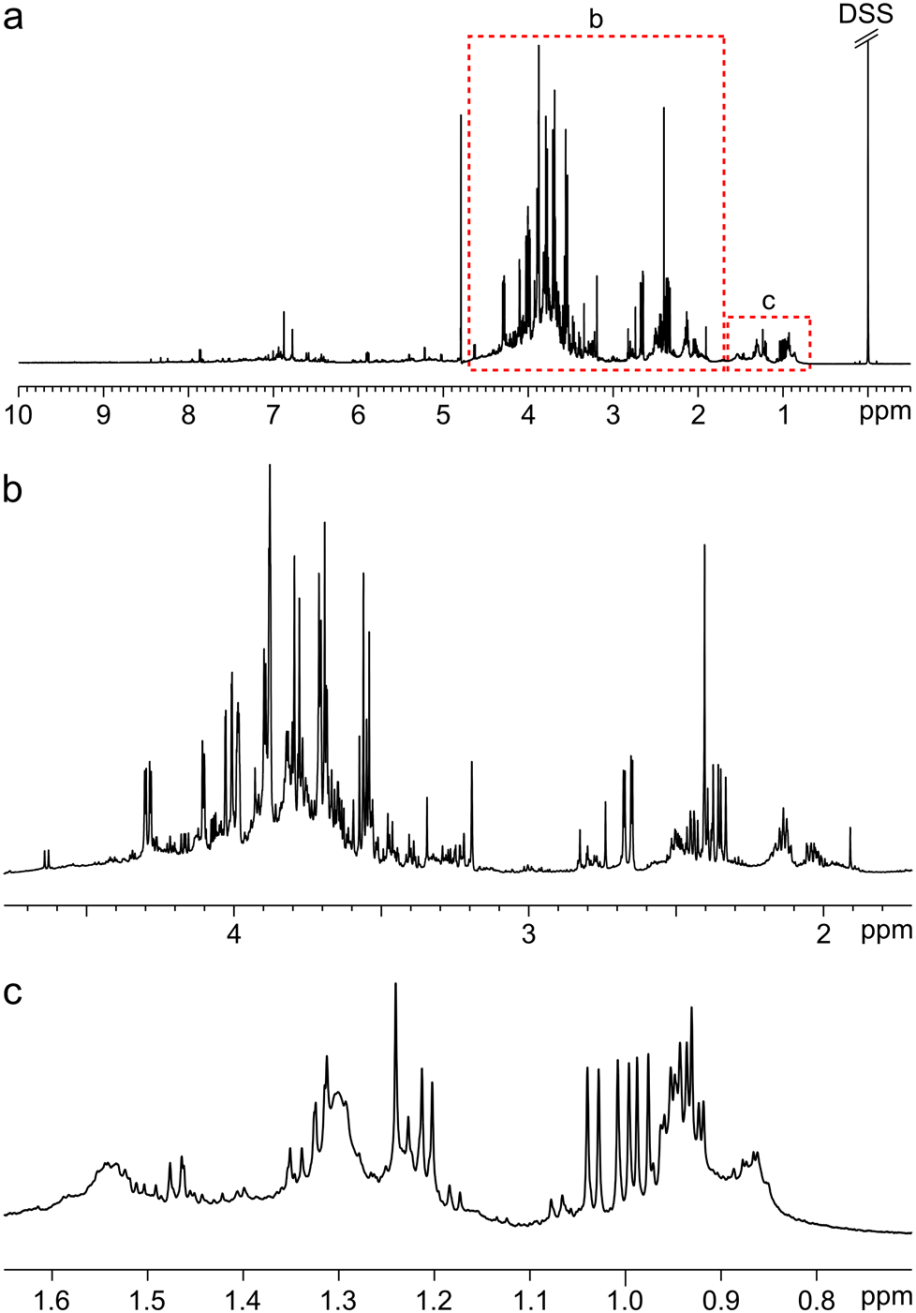
**a** Typical ^1^H NMR spectrum of oilseed rape root exudate dissolved in D_2_O, **b** Magnification of spectral region 1.7–4.8 ppm, **c** Magnification of spectral region 0.70–1.65 ppm

Whereas human blood plasma and serum are well studied by NMR and the majority of signals have been assigned (Psychogios et al., 2011; Nagana Gowda et al., 2015), many other biological samples are less well characterised. Plant samples, for example, are very complex with numerous different metabolites of widely different concentrations, which complicates NMR analysis (Deborde et al., 2017). In the present study, aqueous oilseed rape (*Brassica napus*) root exudate samples were used as a model system to develop the proposed workflow (Fig. 1). Root exudates consist of all substances that are excreted by plant roots during growth, including sugars, organic acids, and amino acids (Vives-Peris et al., 2020). In addition, the samples used in this study all contained various unknown compounds, likely lipids, that gave rise to broad signals in the spectra (Fig. 1b and c). Before accurate quantification can be performed, these signals need to be accounted for in some way.

In blood plasma and serum, macromolecules giving rise to broad signals are routinely removed by ultrafiltration or precipitation with organic solvents before NMR analysis (Daykin et al., 2002; Nagana Gowda & Raftery, 2014). Other options are to use certain NMR experiments that target broad signals, such as the Carr-Purcell-Meiboom-Gill (CPMG) pulse sequence (Carr & Purcell, 1954; Meiboom & Gill, 1958) or diffusion-edited experiments (Liu et al., 1996; de Graaf & Behar, 2003; Bliziotis et al., 2020). There are also methods solely based on computations, such as the Small Molecule Enhancement Spectroscopy (SMolESY) method (Takis et al., 2020, 2021) that utilises the first derivative of the imaginary part of the NMR data to generate a spectrum devoid of broad signals. SMolESY is capable of performing automated relative quantification in blood samples, but for more complex spectra remaining metabolite signal interferences may appear. Because the NMR signals in a SMolESY spectrum are not Lorentzian shaped, standard spectral libraries cannot be used to model these interferences to obtain absolute concentrations. Another strategy is to include broad signals in the quantification methods, either by modelling them as signals using e.g. wavelets (Hao et al., 2012) or Lorentzians (de Graaf et al., 2015), or by treating them as baseline distortions and removing their interference by approximating a baseline correction function through the broad signal (Zheng et al., 2011; Jacob et al., 2017). Most of these methods are developed for plasma, but could potentially also be applied to the plant root exudate samples used as test system in the current study.

The aim of the current study was to develop a rapid, straightforward, and computationally efficient extension of AQuA for absolute quantification of selected metabolites in highly complex spectra containing broad background signals. The method should require minimal sample preparation without compromising the quantitative accuracy. Because of speed and computational cost, we decided to remove the interferences caused by the broad signals before the AQuA computation. This was done using an automatic baseline correction function; here we employed the widely used adaptive iteratively reweighted penalised least squares (airPLS) algorithm (Zhang et al., 2010). The combined method, called extended AQuA, was evaluated using simulations as well as a spike-in experiment performed in a complex sample matrix. This showed that the approach is both accurate, linear, and robust. Furthermore, the proposed workflow is fast and flexible and can easily be fine-tuned for individual samples.

## Materials and methods

### Root exudate collection

Seeds of various spring varieties of oilseed rape (*Brassica napus*) were kindly provided by Scandinavian Seed AB and Lantmännen Seed AB. All glassware was rinsed extensively with MilliQ water and autoclaved before use to minimise traces of detergents. Seeds were surface sterilised (10% chlorine bleach for 5 min with mild shaking) and then rinsed with autoclaved MilliQ water four times. The seeds were germinated on petri dishes containing 0.5× Murashige-Skoog medium, including vitamins (MS0222, Duchefa Biochemie B.V., Haarlem, Netherlands) and 0.6% bacto agar, in a growth chamber at 22/20 °C (day/night), 16/8 h photoperiod with 110 µE. After three to five days of germination, when cotyledons and rootlets were expanded, plantlets (*n* = 8) were transferred to sterile plastic nets attached to 50 ml plastic tubes filled with autoclaved MilliQ water, so that the seedling roots were immersed into the water. This procedure was done in a sterile laminar flow hood. The samples were placed in a sterilised transparent plastic box and kept for four days with slow agitation in a growth chamber at 22/20 °C (day/night), 16/8 h photoperiod with 110 µE. Exudates were collected into glass bottles in a sterile laminar flow hood, shell frozen and lyophilised in darkness. Aliquots of the exudates were spread on plates containing LB agar or 0.5× Murashige-Skoog agar and stored for 48 h to assess any microbial contamination. Blank samples did not contain any seedlings but were otherwise treated as described above.

Lyophilised root exudate and blank samples were dissolved in a few millilitres of MilliQ water, transferred to 15 ml plastic tubes, and dried in a vacuum centrifuge. Dried samples were stored in a desiccator until use.

### Sample preparation

NMR samples were prepared in a similar fashion to a previously published protocol (Kim et al., 2010). All experimental work was performed at room temperature. 750 µl KH_2_PO_4_ buffer in D_2_O (45 mM, pD 7.0 (apparent pH 6.6) containing approximately 0.29 mM DSS-*d*_6_ (sodium 3-(trimethylsilyl)propane-1-sulfonate-*d*_6_) was added to each sample. The samples were vortexed 30 s followed by 10 min ultrasonication. This procedure was repeated once. The samples were then transferred to 1.5 ml plastic tubes and centrifuged for 10 min at 17 000×*g*. For each sample, 600 µl of the supernatant was added to a 5 mm NMR tube.

### NMR spectroscopy and spectral processing

NMR spectra were acquired on a Bruker Avance III 600 MHz spectrometer with a 5 mm ^1^H/^13^C/^15^N/^31^P inverse detection cryoprobe equipped with a z gradient. 1D-^1^H NMR spectra (256 transients) were recorded at 25 °C using a NOESY presaturation pulse sequence (Bruker’s *noesypr1d*) with 1 s relaxation delay, 100 ms mixing time, 4.5 s acquisition time, and 12 ppm spectral width, to enable absolute quantification based on the Chenomx library. 65 536 data points were collected and the carrier frequency was placed on the HDO signal (4.70 ppm). After acquisition, an exponential line broadening of 0.3 Hz was applied and the spectral quality was evaluated by assessing the full width half maximum (FWHM) of the DSS signal. If FWHM_DSS_ was greater than 1.20 Hz, a new spectrum was recorded. Spectra were processed (zero-filling, line broadening, phase correction, crude baseline correction) using Chenomx NMR Suite Professional Software package (version 8.6, Chenomx Inc., Edmonton, Canada). The line-broadening factor was adjusted for each spectrum to obtain FWHM_DSS_ = 1.20 Hz. If necessary, a crude baseline correction was applied to obtain a flat baseline around the internal standard signal before determining FWHM_DSS_. The processed spectra were subjected to spectral binning (− 0.50 to 4.68 ppm and 4.98 to 10.00 ppm, 0.0002 ppm/bin, 51 000 bins in total) and imported to MATLAB (version R2020a, MathWorks Inc., Natick (MA), USA).

To verify metabolite identification, ^1^H,^1^H-TOCSY (Bruker pulse sequence *dipsi2gpphpr*) and ^1^H,^13^C-HSQC (Bruker pulse sequence *hsqcedetgpsisp.2*) spectra were recorded for some of the samples. These spectra were processed with TopSpin 4.0.6 (Bruker BioSpin).

### Metabolite identification and quantification

AQuA does not attempt at automated metabolite identification, hence metabolite signals have to be selected prior to AQuA computation. Here, identification of metabolites was based on previous literature (Vives-Peris et al., 2020) and reference NMR spectra included in the Chenomx library. The identity of the metabolites was verified with ^1^H,^1^H-TOCSY and ^1^H,^13^C-HSQC NMR spectra recorded for some of the samples. ^13^C NMR chemical shifts were compared with those available in the Biological Magnetic Resonance Data Bank (Ulrich et al., 2008). Only metabolites verified by 2D NMR experiments, or displaying an excellent fit for several signals with the Chenomx library, were included in the quantification model. Furthermore, only primary metabolites were included since one of the aims was to develop a method capable of quantifying only a subset of all metabolites in an NMR spectrum.

Binned processed NMR spectra (see Sect. 2.3) were imported to MATLAB and subjected to the airPLS algorithm (Zhang et al., 2010) to fine-tune the baseline where affected by irregularities or the presence of broad signals. As default, the airPLS smoothing factor λ was set to 1 × 10^7^, but a local value was determined for spectral regions where the default λ failed to yield a satisfactory baseline correction. The values for the other parameters in the airPLS algorithm were used as default (order = 2, weight exception proportion = 0.1, asymmetry parameter = 0.05, and maximum iteration time = 20). The airPLS algorithm, using the optimised λ values, was incorporated in an automated joint workflow with AQuA in MATLAB. This workflow is referred to as the extended AQuA. Metabolite quantification using AQuA was performed on the corrected spectra according to the strategy previously described (Röhnisch et al., 2018), using the Chenomx library as a basis to model metabolite signals. In total, 24 metabolites were targeted for quantification, including various amino acids, organic acids, and sugars (Table S1). One reporter signal to be used for quantification was selected for each metabolite (Table S1). Additionally, a few unknown signals were included in the model as Lorentzians generated in Chenomx (Fig. S1).

### Simulations

A simple smoothing algorithm developed in-house was applied to one root exudate spectrum to model the spectral background. The algorithm was built in MATLAB based on the ‘smooth’ function. In short, the following steps were employed: (1) localisation of narrow high-intensity signals (spikes), (2) determination of spike borders, (3) spike depletion by linear regression inside spike borders, and (4) average-based smoothing of the spike-depleted spectrum (for more information and a visual description of the process, see Supplementary Information Sect. 3, especially Fig. S5). In the final step, three levels of smoothing (low, medium, and high) were used to obtain three distinct spectral background models (referred to as A, B, and C, respectively, see Fig. S6). Normalised reference spectra of 24 metabolites (Table S1) were summed together and added to each spectral background in seven different scaling levels, thus yielding 21 simulated spectra. The spectra were corrected with the airPLS algorithm using three different λ values (1 × 10^6^, 1 × 10^7^, and 1 × 10^8^) applied to the whole spectra. Peak picking of one signal per metabolite was performed as previously described (Röhnisch et al., 2018) to obtain signal intensities in the corrected spectra.

### Spike-in experiment

Six of the analysed root exudate samples were pooled together and then divided into five portions. Five metabolites (γ-aminobutyric acid (GABA), dl-asparagine, l(+)-tartaric acid, L-threonine, and D-xylose) not present in the pooled sample were added to different concentrations. As control, five identical blank samples were spiked the same way. The chosen metabolites have signals in different spectral regions with different multiplicities and differ in how much they are affected by broad signals or baseline distortions. The large variation in concentration (10 µM-3200 µM) between the spiked metabolites reflects the large dynamic range observed in the experimental data set, both between different metabolites in the same sample and between the same metabolite in different samples. See Supplementary Information, Sect. 4.1, for more details about the design of the spike-in experiment.

The spiked root exudate samples were analysed as described above, i.e. NMR analysis, spectral processing, and metabolite quantification using an airPLS-extended AQuA, which had been adjusted to include all spiked metabolites (Table S1).

The spectra of the spiked blank spectra were carefully baseline corrected in Chenomx. For the analysis of these spectra, the airPLS step was omitted and an AQuA that only targeted the five spiked metabolites plus lactic acid was used to calculate metabolite concentrations.

## Results and discussion

### Extended AQuA: workflow, parameter optimisation, and general considerations

The 1D-^1^H NMR spectra of oilseed rape root exudates displayed baseline irregularities, including broad background signals, that would impair metabolite concentration estimates if not properly accounted for (Fig. 1). The broad signals in the low-frequency part of the spectra were the most problematic distortions, due to their interference with several amino acid signals. Different methods for elimination of the baseline distortions were evaluated, utilising sample preparation, spectral editing, and computations, respectively (see Supplementary Information, Sect. 2). It was found that an automatic baseline correction function such as the airPLS algorithm (Zhang et al., 2010) could be employed to yield root exudate spectra suitable for targeted metabolomics, i.e. with well-preserved metabolite signal line shapes, a flat baseline, no pronounced residual broad signals, and no severe intensity modulation (see Fig. S2). Manual baseline correction was not considered feasible due to the complexity of the spectra.

**Fig. 2:**
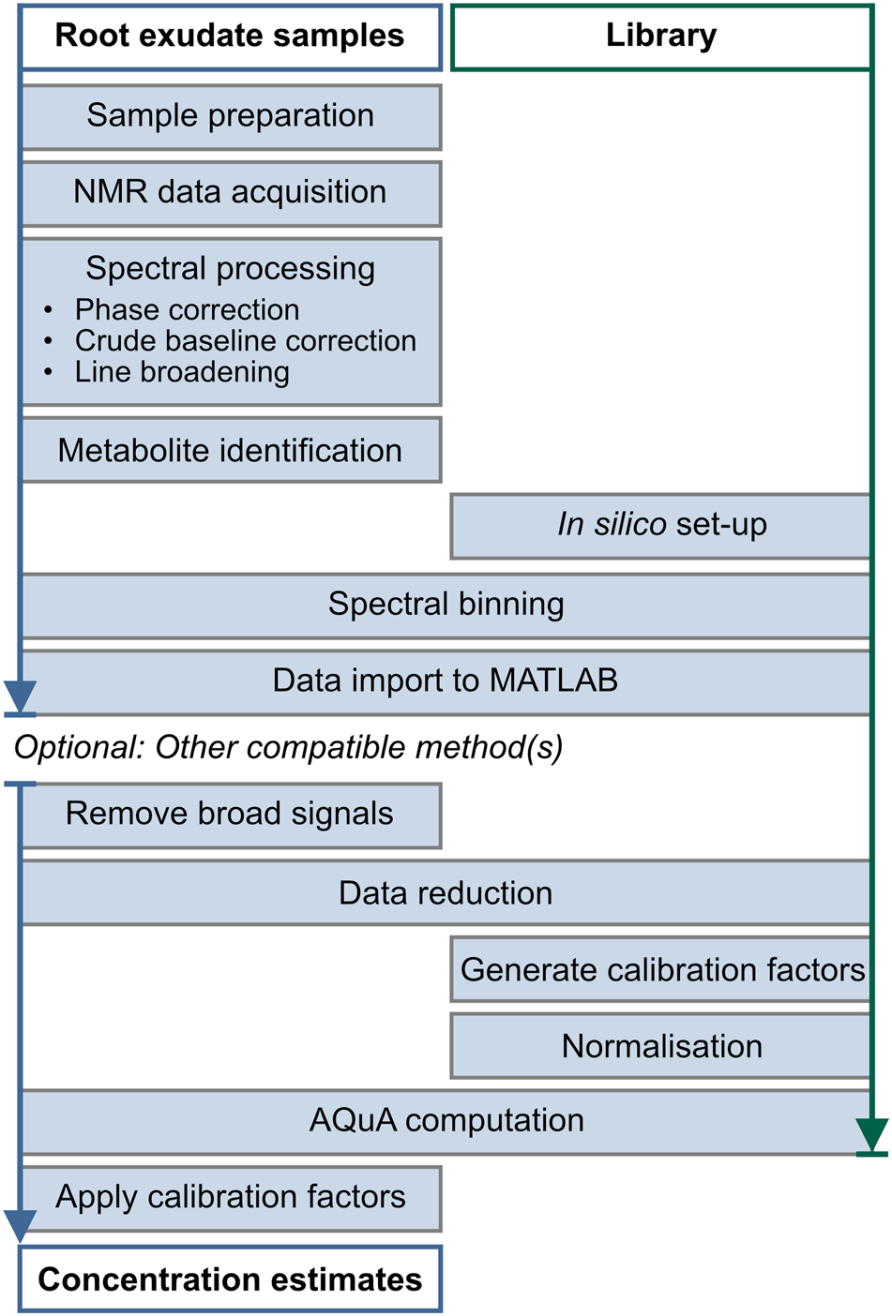
Proposed workflow for NMR-based quantification of primary metabolites in plant root exudates after minimal sample preparation (see Materials and methods). The workflow includes the airPLS algorithm (Zhang et al., 2010) for removal of broad signals, followed by quantification using AQuA (Röhnisch et al., 2018). The *in silico* library, here created from Chenomx, contains 1D-^1^H NMR spectra of all targeted metabolites. Other compatible methods, e.g. for signal alignment, can also be included in the workflow if desired

The airPLS algorithm was combined with AQuA into a joint automated workflow, i.e. the extended AQuA, for quantification of metabolites in experimental ^1^H NMR spectra of root exudates, acquired with minimal prior sample preparation (Fig. 2). The identity of the metabolites was confirmed with ^1^H,^1^H-TOCSY and ^1^H,^13^C-HSQC experiments. Because the AQuA quantification is based on just one signal per metabolite, the airPLS algorithm was used to obtain a good baseline around these signals only, rather than aiming for a perfect baseline in the entire spectrum. The spectral library used here was created from Chenomx but other sources, e.g. in-house libraries, can be used instead if desired.

**Fig. 3:**
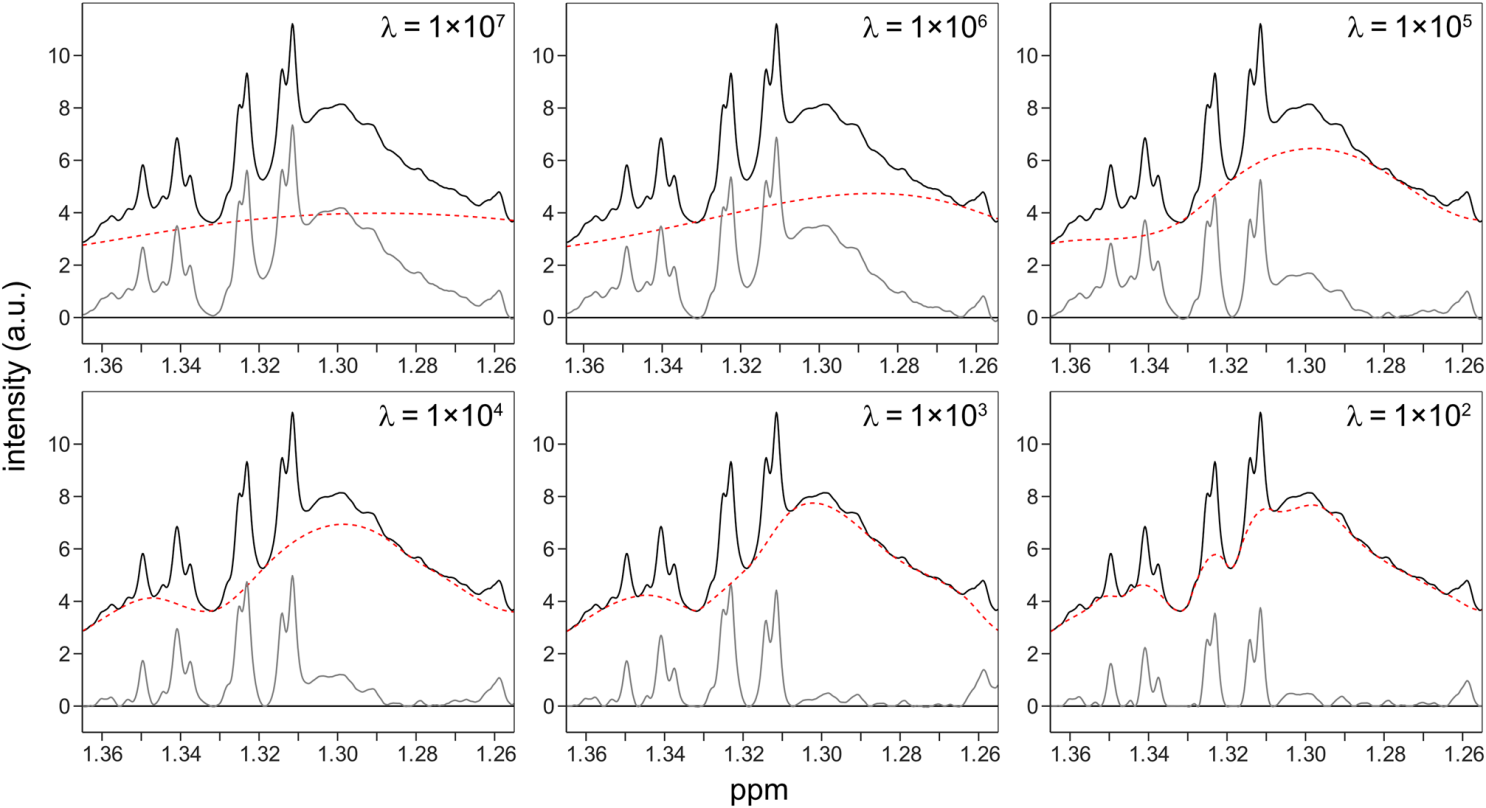
The effect of different λ values (1 × 10^7^ − 1 × 10^2^) on the baseline correction of the lactic acid/threonine region of a root exudate spectrum. Black: experimental spectrum before baseline correction, dashed: fitted baseline, grey: experimental spectrum after baseline correction. Note that the experimental and corrected spectra are superimposed, not stacked on top of each other

For the airPLS algorithm to work properly, the smoothing factor λ needs to be optimised. This parameter, which can be set to any value between 1 and 1 × 10^9^ (Zhang et al., 2010), strongly affects the result of the baseline correction. If λ is set too high, the fitted baseline does not include enough of the background, whereas if it is set too low, the algorithm starts to remove parts of the metabolite signals (Fig. 3). Here, due to the non-uniform distribution of broad signals and other baseline distortions, a single λ value was not used for an entire spectrum; instead, different λ values were used for different spectral regions (see Sect. 3.2). Despite the virtually unlimited number of options, it was neither difficult nor time-consuming to find suitable λ values. Importantly, the optimised λ values could be kept fairly constant throughout each data set and could thereby be included in the automated workflow. Before applying the extended AQuA to a data set, the result of the baseline correction should be assessed carefully on a representative subset of the spectra, although one has to keep in mind that the procedure is inevitably an estimation and may not exactly match the actual baseline of the spectrum. However, this is true for all baseline correction methods, regardless of if they are manual or automated.

In addition to baseline distortions, interference can also be caused by spectral overlap with narrow unknown signals. In the current study, the aim was to quantify a preselected subset of metabolites while leaving remaining signals in the spectra untargeted. However, other signals that interfere with the metabolite signals used in AQuA need to be included in the quantification model to avoid overestimating the metabolite concentrations. Here, four unknown signals between 0.93 and 0.97 ppm were added to the quantification model as single Lorentzians to obtain a more accurate concentration estimate of leucine based on the signal at 0.96 ppm (Table S1 and Fig. S1).

### Evaluation of the extended AQuA

#### Simulations

**Fig. 4:**
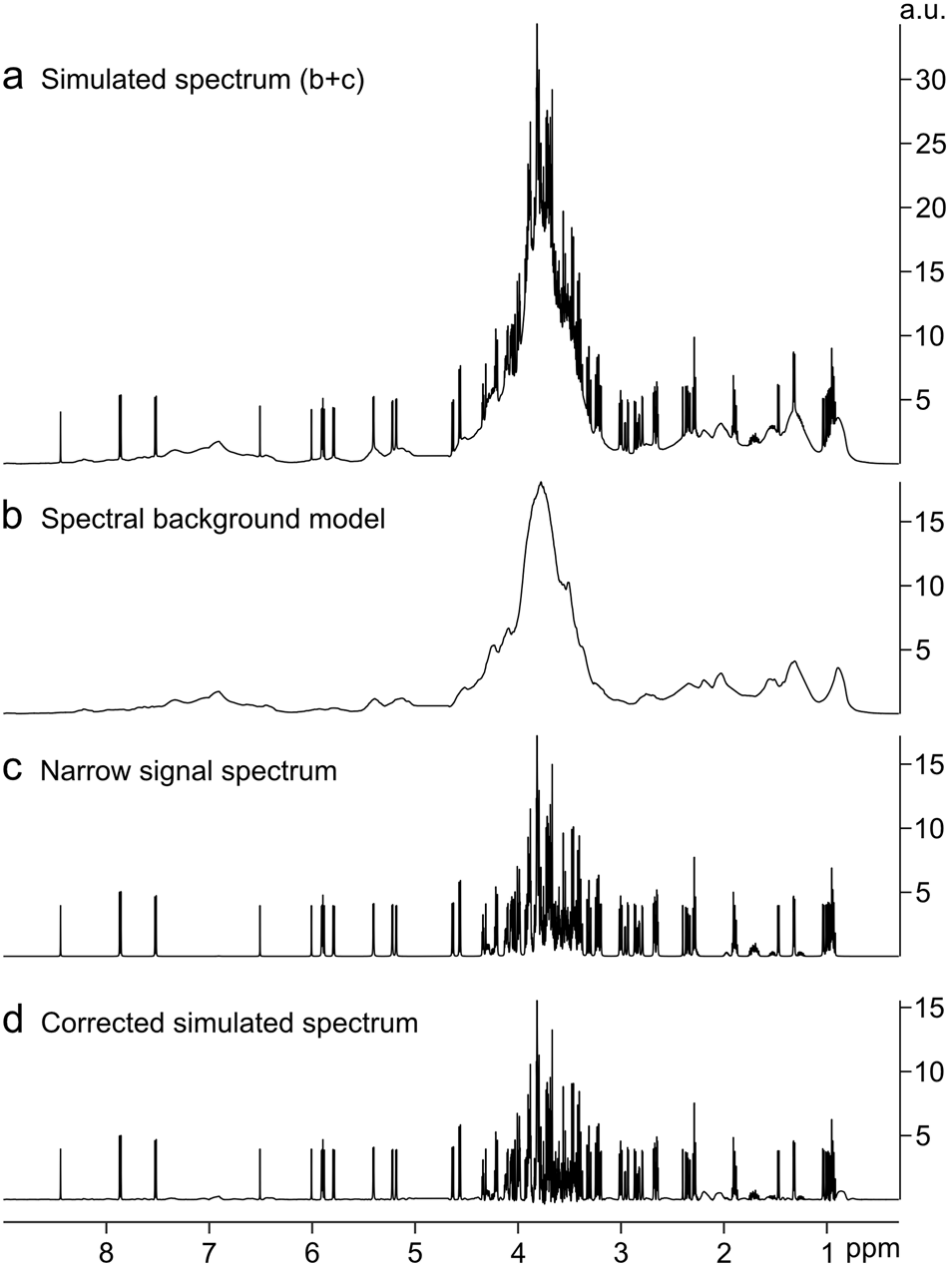
Examples of simulated spectra used to evaluate the extended AQuA. **a** Simulated root exudate spectrum, constructed from a simulated spectral background **b** and a simulated narrow signal spectrum **c**. **d** The simulated root exudate spectrum after correction with the airPLS algorithm (λ = 1 × 10^7^ for the entire spectrum). Ideally, the spectra in **c** and **d** should be identical. An intensity scale has been added to all spectra to facilitate comparison between the spectra

The extended AQuA was first evaluated using simulated spectra of root exudates where the contributions of the broad signal background and the narrow metabolite signals were exactly known (Fig. 4). To test how well the method can handle different types of spectra, three different spectral background models (A, B, and C) with varying smoothness were created (Figs. S5 and S6) and a simulated narrow signal spectrum was added to the backgrounds in seven different intensity levels. In total, 21 simulated spectra were thus obtained with differences in their spectral backgrounds as well as in their ratio between narrow and broad signals (Figs. S7–S10 and Table S2). For reference, Fig. 4a depicts the simulated spectrum created with the medium-smooth background B (Fig. 4b) and an intermediate intensity of the narrow signal spectrum (Fig. 4c). The airPLS algorithm was applied three times to all spectra, with three different λ values, to evaluate the robustness of the method. The signal heights in the airPLS corrected spectra (Fig. 4d) were compared to those in the corresponding narrow signal spectra (Fig. 4c) using linear regression. Thereby, it was possible to precisely assess how well the airPLS algorithm could remove interferences caused by broad signals and baseline irregularities, and to what extent the narrow signal part of the spectrum was affected by the procedure.

**Fig. 5:**
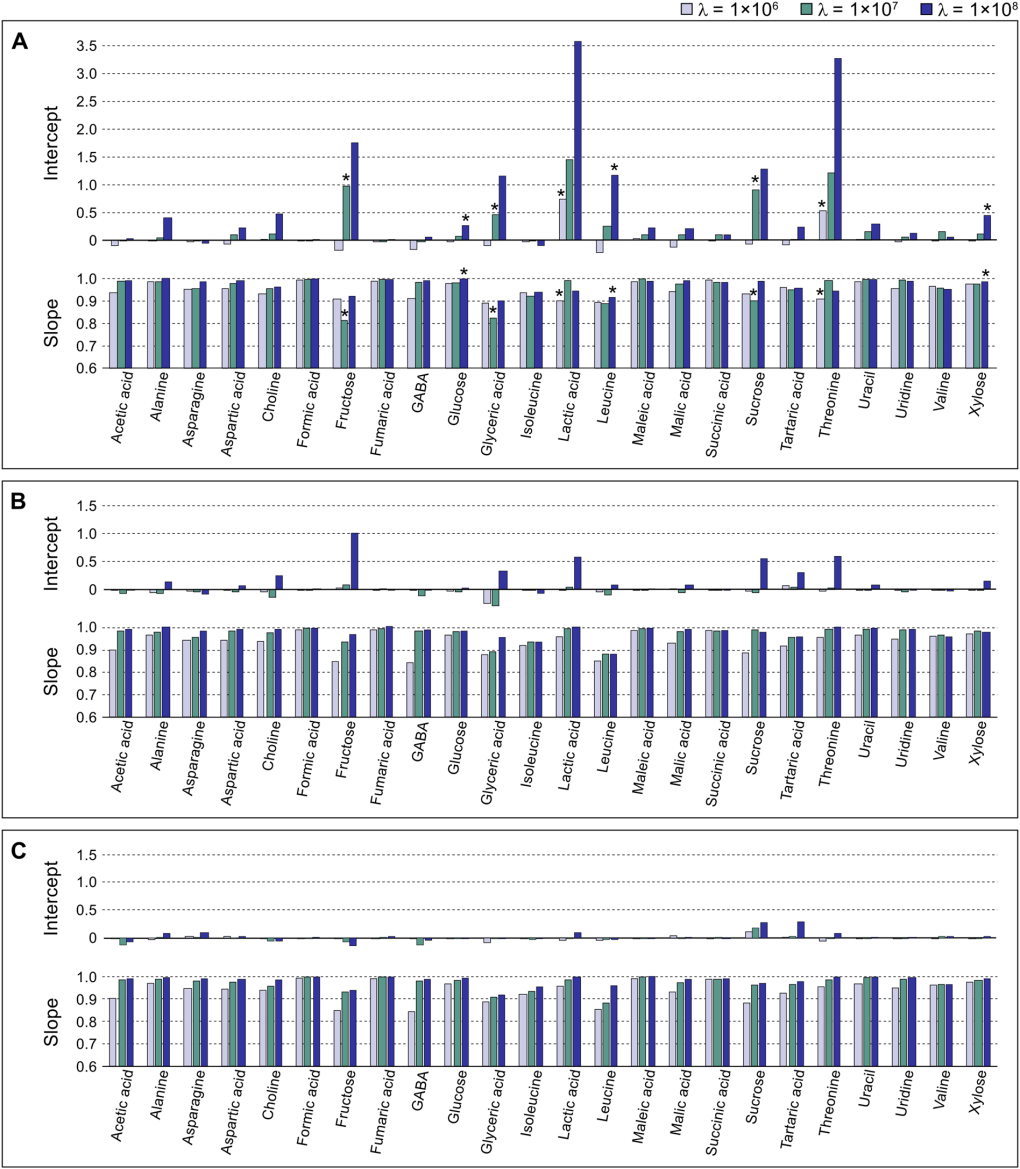
Evaluation of the extended AQuA applied to seven simulated spectra constructed using spectral background model A, B, or C (see Figs. S5–S9). Linear regression was performed using signal heights in the simulated narrow signal spectra as predictor (x-axis) and signal heights in the corresponding simulated spectra with both broad and narrow signals, after correction with the airPLS algorithm, as response (y-axis). Only the signals used in AQuA were evaluated. Bar heights display values of the intercepts and slopes for each metabolite in the simulated spectra. Bar colour indicates the λ value used in the baseline correction. Asterisks denote linear regressions with R^2^ < 0.9900

In general, the agreement between the intensities in the baseline corrected spectra and the original narrow signal spectra was good for the signals used in AQuA, as indicated by slopes and R^2^ coefficients close to one and intercepts close to zero (Fig. 5 and Table S3). This suggests that the airPLS feature specifically corrected the baseline and removed broad background signals without notably affecting the selected metabolite signals. Percentage differences (Table S3) were calculated to condense the accuracy estimate into a single variable. For most metabolites, the difference was less than 10% with at least one of the λ values. The smoother backgrounds B and C were easier to fit than the rougher background A, hence the smaller intercepts (Fig. 5b and c). Overall, when the airPLS algorithm was applied to spectra created using background A the λ value needed to be smaller than for spectra based on background B or C. For metabolite signals situated in spectral regions without background interference, e.g. formic acid and fumaric acid, the accuracy was good for all spectra regardless of which λ value was being used (Fig. 5 and Table S3). In contrast, some metabolites had signal intensities in the corrected spectra that deviated substantially from their true values. Often, this coincided with a pronounced interference from the spectral background (see Table S2 and Fig. S10). For example, the signals of fructose, glyceric acid, lactic acid, and threonine were all highly influenced by the spectral background and so the quantification accuracy of these metabolites was strongly dependent on the performance of the baseline correction. Because of the large variation in signal intensity in the simulated spectra, the results could have been more accurate if the λ value had been optimised for each individual spectrum (see next section). However, in an experimental data set, the inter-spectral variation is usually not as big. Furthermore, the quantification accuracy for a given metabolite generally increased with increasing signal intensity relative to the spectral background. Thus, the lower the intensity of the narrow signals and the higher the intensity of the background, the more critical it becomes to optimise the method parameters to avoid quantification errors. Ideally, the signals used in the AQuA computation should all have a low degree of interference and high signal to noise ratio (Röhnisch et al., 2018); however, this is not possible for all metabolites. Still, the proposed method appears to be both linear and accurate for most metabolites.

#### Spike-in experiment

A spike-in experiment was conducted to further evaluate the extended AQuA (Tables S4, S5 and Fig. S11). Five metabolites (asparagine, GABA, tartaric acid, threonine, and xylose) were added both to blank samples and to aliquots of a pooled root exudate sample in concentrations above the limit of quantification (10 × S/N) for each metabolite. The blank spectra displayed minimal signal interference and lacked the broad background signals and baseline distortions that were present in the root exudate spectra. Therefore, these spectra were only subjected to manual baseline correction before the AQuA computation. The spiked root exudate spectra, on the other hand, were baseline corrected with the airPLS algorithm to remove broad background signals. Here, the default λ value gave a satisfactory correction for all metabolites except threonine and GABA, as evaluated by manual inspection. Threonine was the most challenging metabolite to quantify in the spiked root exudate spectra because its selected signal overlapped both with the signal of the methyl group of lactic acid and with a broad signal that was not assigned unambiguously but can be tentatively attributed to a lipid methylene signal (Fig. S11). The latter could not be correctly suppressed unless a lower λ value was used (see Fig. 3). The GABA signal is a broad quintet whose intensity was slightly reduced with the default λ value because the fitted baseline removed a small portion of the signal (Fig. S12). Therefore, the size of λ was increased for this spectral region.

After baseline correction, AQuA computation was performed on both the spectra from the spiked root exudates and the spiked blank samples, and the results were compared with each other using linear regression as well as percent differences (Table 1). The calculated concentrations are listed in Table S6. Because the same amount of metabolites were added to both sample sets, all slopes should theoretically be equal to one, and all intercepts should be equal to zero as none of the spiked metabolites were present in the samples initially. However, since the sample matrices differed somewhat and all metabolite additions were done manually, some deviations could be expected. Still, as shown in Table 1, the R^2^ values were > 0.999, all intercepts were close to the origin, and the percent differences were generally small. This was in agreement with the results from the simulations. To enable comparison of the intercepts amongst the different metabolites despite the big differences in concentration, the intercepts are reported both as the actual value and as percent of the highest concentration for each metabolite. The tartaric acid signal was consistently more intense in the spectra of the spiked root exudate samples than in the spectra of the corresponding blank samples (Fig. S13), hence the large slope and percent differences. An experimental error probably occurred when tartaric acid was added to the root exudate samples since the calculated concentration of tartaric acid in the blank samples, but not the root exudate samples, agreed well with the actual concentrations (Tables S6–S8). The values of the intercepts and slopes for the other metabolites indicated that there was no clear, systematic over- or underestimation of the concentrations obtained using the proposed method compared to when the same metabolites, in the absence of baseline distortions, were quantified with the non-extended AQuA.

**Table 1:** Comparison of the concentrations obtained for the spiked blank samples and the concentrations obtained for the spiked root exudate samples^a^

Metabolite	Max conc. (µM)^b^	R^2^	Slope	Intercept (µM)	Rel. intercept^c^ %	Mean % difference blank-sample^d^
Asparagine	1604	1.0000	0.976	− 0.474	− 0.0295	2.7
GABA	403	1.0000	1.00	− 3.23	− 0.801	4.0
Tartaric acid	800	0.9994	1.16	6.70	0.837	20.0
Threonine	164	0.9996	1.01	2.22	1.36	6.2
Xylose	3224	1.0000	0.999	4.49	0.139	1.2

^a^Results from linear regression. Predictor (x-axis): Concentrations for spiked blank samples calculated using an AQuA including only the five spiked metabolites and lactic acid. Response (y-axis): Concentrations for spiked root exudate samples calculated using an airPLS-extended AQuA including the metabolites listed in Table S1. Used airPLS parameters: λ_default_ = 1 × 10^7^, λ_Thr_ = 1 × 10^5^ − 1 × 10^6^ (depending on the intensity of the threonine signal), λ_GABA_ = 1 × 10^8^

^b^Actual value for the spiked sample with the highest concentration

^c^Intercept as percent of the maximum concentration for the metabolite

^d^Average difference (%) for each spiked metabolite, comparing the calculated concentrations found for the spiked root exudate samples with the calculated concentrations for the corresponding blank samples (calculated as 100 × |C_blank_ − C_sample_|/C_blank_). See Table S6 for a complete list of difference values

### Application to plant root exudates

The extended AQuA was applied to a data set consisting of 50 NMR spectra from oilseed rape root exudates and 7 blank spectra. Concentration estimates were computed for 24 metabolites (Table S1). Additionally, four unknown signals were included to model signal interferences (Fig. S1) but they were not quantitatively interpreted.

The extended AQuA process (i.e. baseline correction followed by the quantification of 24 target metabolites) applied to all 57 spectra was typically completed in less than 30 s on a standard personal computer. The same method parameters were used for all spectra. In addition to the default λ value, two local values were used in the airPLS baseline correction (λ = 1 × 10^6^ for the spectral region 0.899–0.967 ppm and λ = 1 × 10^5^ for the region 1.225–1.334 ppm). If only one λ value was used, the total computation time decreased to around 10 s. It has been shown that AQuA requires less than one second to quantify 67 metabolites in 1342 spectra (Röhnisch et al., 2018). Introducing the airPLS step thus increases the computation time but the combined method is still very rapid. Because the airPLS algorithm is the rate-limiting step, the computation time increases notably with the number of spectra and λ values whereas it is negligibly affected by the number of metabolites targeted for quantification.

### Advantages and limitations

The method described here allows for quantification of metabolites in complex spectra that contain broad signals and baseline distortions. Only minimal sample preparation is required and because the method is purely computational, knowledge about the compounds causing the broad signals is not needed. However, in case of binding interactions between metabolites and other compounds such as proteins, application of the method would be more challenging. The occurrence of such interactions can be estimated by assessing the line width and shape of the internal standard signal, since both DSS and TSP are known to interact with macromolecules (Bell et al., 1989; Kriat et al., 1992; Shimizu et al., 1994; Nowick et al., 2003). Here, both metabolite signals and the internal standard signal were narrow and symmetric, which indicated that no significant macromolecular interaction was taking place.

As shown here, the baseline correction method airPLS and the quantification method AQuA can be combined into a fully automated workflow, provided that prior metabolite identification and parameter optimisation have been conducted. Optimising the airPLS algorithm is straightforward and depends only on the parameter λ. Here, we did not strive for an optimal baseline in the whole spectrum but only in regions containing signals used in AQuA, which reduced the optimisation time and effort. The combined method is extremely fast and typically requires less than one second per spectrum. This is due both to the sparse matrix characteristic of the airPLS algorithm, but more importantly the AQuA data reduction strategy. AQuA only considers a set of pre-selected signals in the quantitative process, one for each metabolite, which facilitates very rapid computations whilst still accounting for interferences between metabolites.

Here, we chose to use the airPLS algorithm for baseline correction but it is possible to use other methods instead, as long as they are compatible with AQuA. The metabolite library can also be exchanged if e.g. in-house spectral libraries are preferred.

There are also some limitations of the method. Relying on the height of one single metabolite signal for deriving concentrations may make the method more sensitive to systematic errors caused by database discrepancies compared to when several signals are used (see Supplementary Information, Sect. 4.2). However, this has not been fully evaluated, neither have we investigated whether other quantification methods are less susceptible to this kind of errors. Since AQuA is not an identification method, there is also a risk of erroneous metabolite quantification if the chemical shift windows have not been properly selected or if there are unknown signals present in some spectra that have not been accounted for. If a signal from another compound, metabolite or impurity, with higher intensity than the intended metabolite signal resides in the chemical shift window, the algorithm will pick this signal for quantification instead. For reliable results, metabolite identification should ideally be assessed manually. However, the problem with possible false identification of metabolites is not unique to AQuA, especially when the targeted metabolite signals are singlets.

## Conclusions

We have here presented a fast and accurate approach for automated quantification of selected metabolites in complex NMR spectra. The spectra of minimally handled plant root exudate samples were successfully analysed with the proposed method, despite the presence of unknown broad signals, baseline distortions, and extensive spectral overlap. Although not evaluated here, the method is theoretically applicable to any spectrum with similar characteristics, as long as the metabolite signals are unaffected by macromolecular interactions.

### Supplementary Information

Below is the link to the electronic supplementary material.

Supplementary material 1 (PDF 2820.5 kb)

## Data Availability

The NMR data has been uploaded to the Swedish National Data Service and is available at 10.5878/8t82-d090.
