# Supplementary material for: Extended automated quantification algorithm (AQuA) for targeted 1H NMR metabolomics of highly complex samples: application to plant root exudates
Source: Metabolomics. 2023 Dec 23;20(1):11. doi: 10.1007/s11306-023-02073-z (PMC10748781; doi:10.1007/s11306-023-02073-z)
Supplement: Supplementary file 1 — Supplementary material 1 (PDF 2820.5 kb) [file 11306_2023_2073_MOESM1_ESM.pdf]

Supplementary information

Extended Automated Quantification Algorithm (AQuA) for targeted ^{1}H NMR metabolomics of highly complex samples: Application to plant root exudates

Elin Alexandersson[1*], Corine Sandström[1], Johan Meijer[2], Gustav Nestor[1], Anders Broberg[1] & Hanna E. Röhnisch[1]

[1] Department of Molecular Sciences, Swedish University of Agricultural Sciences, Uppsala, Sweden
[2] Department of Plant Biology, Swedish University of Agricultural Sciences, Uppsala, Sweden

* Corresponding author. E-mail: elin.alexandersson@slu.se

Table of content

1. Metabolite library………………………………………………………………………...S3

Table S1: The metabolites included in AQuA applied to the different data sets…..................S3

Fig. S1: Overlap with unknown signals…………………………………….........................S4

2. Evaluation of different methods for suppression of broad signals in 1D-^{1}H NMR spectra……S5

2.1 Evaluation results………………………...…………………...…………………………..S5

Fig. S2: Ultrafiltration, SPE, CPMG, 1D-diffusion, and airPLS applied to the same root exudate sample……………………………………………………………………………......S6

Fig. S3: 1D-^{1}H NMR spectra of a pooled root exudate sample either analysed directly or after it was passed through an SPE column………………………………………………………S7

Fig. S4: 1D-NOESY-presat, 1D-diffusion, and the difference spectrum of a pooled root exudate sample……………………………………………………………………………......S8

2.2 Sample preparation and acquisition parameters……………………………………………..S9

3. Simulated spectra……………………………………………………………………...S10

Fig. S5: Modelling the spectral background of a root exudate spectrum…………………….S10

Fig. S6: The three spectral background models included in the simulations………………….S11

Fig. S7: The seven simulated spectra based on background A………………………………..S12

Fig. S8: The seven simulated spectra based on background B………………………………..S13

Fig. S9: The seven simulated spectra based on background C……………………………..…S14

Fig. S10: The metabolite signals that were targeted in the analysis of the simulated spectra……………………………………………………………………………..S15

Table S2: The contribution of the spectral background to the total target signal intensities in the simulated root exudate NMR spectra……………………………………………..…..S18

Table S3: Evaluation of the extended AQuA applied to 21 simulated NMR spectra………..…..S19

4. Spike-in experiment……………………………...…………………………………….S21

4.1 Methodology………………………………………………………………………......S21

Table S4: Summary of the target signal characteristics of the spiked metabolites……………S21

Table S5: Approximate concentrations in the samples after spiking with 5 µl of each standard solution…………………………………………………………………………….S21

Fig. S11: NMR spectra of the pooled root exudate sample before and after addition of the spike-in metabolites…………………………………………………………………………S22

Fig. S12: The effect of two different λ values on the baseline correction around the GABA signal used in AQuA……………………………………………………………………………S23

Fig. S13: The signal of tartaric acid in the spectra of the spiked root exudate samples overlaid with the spectra from the corresponding spiked blank samples……………………………….S23

4.2 Comparison of AQuA results with the actual concentrations……………………………….S23

Table S6: Calculated concentrations from the spike-in experiment……………………………..S24

Table S7: Comparison of the actual spiked concentrations with the AQuA concentrations obtained for the spiked root exudate samples…………………………………………………S25

Table S8: Comparison of the actual spiked concentrations with the AQuA concentrations obtained for the spiked blank samples……………………………………………………...S25

Fig. S14: Experimental spectrum of asparagine, dissolved in either 90% H_2O/10% D_2O or 100% D_2O, overlaid with the Chenomx library spectrum……………………………………...S26

References………………………………………………………………………………...S26

1. Metabolite library

Table S1 The metabolites included in AQuA (indicated by "X") applied to the different data sets

	Signal used in AQuA				Data set			
**Metabolite**	**δ (ppm)**	**mult.**	**no. H**	**structure**	**Root exudates**	**Simulations**	**Spike-in, samples**	**Spike-in, blanks**
Acetic acid	1.91	s	3	$–CH_3$	X	X	X	
Alanine	1.48	d	3	$–CH_3$	X	X	X	
Asparagine	2.86	dd	1	$–CH_2–$	X	X	X	X
Aspartic acid	2.68	dd	1	$–CH_2–$	X	X	X	
Choline	3.19	s	9	$–CH_3$	X	X	X	
Formic acid	8.45	s	1	=CH–	X	X	X	
Fructose	4.10	m	2	>CH–	X	X	X	
Fumaric acid	6.51	s	2	=CH–	X	X	X	
GABA	1.89	m	2	$–CH_2–$	X	X	X	X
Glucose	5.22	d	1	>CH–	X	X	X	
Glutamine	2.46	m	2	$–CH_2–$	X			
Glyceric acid	4.07	dd	1	>CH–	X	X	X	
Isoleucine	1.01	d	3	$–CH_3$	X	X	X	
Lactic acid	1.31	d	3	$–CH_3$	X	X	X	X
Leucine	0.96	m	6	$–CH_3$	X	X	X	
Maleic acid	6.01	s	2	=CH–	X	X	X	
Malic acid	2.68	dd	1	$–CH_2–$	X	X	X	
Pyroglutamic acid	4.17	dd	1	>CH–	X			
Succinic acid	2.40	s	4	$–CH_2–$	X	X	X	
Sucrose	4.22	d	1	>CH–	X	X	X	
Tartaric acid	4.31	s	2	>CH–		X	X	X
Threonine	1.31	d	3	$–CH_3$	X	X	X	X
Unknown-1[a]	0.94	s	-	-	X			
Unknown-2[a]	0.95	s	-	-	X			
Unknown-3[a]	0.96	s	-	-	X			
Unknown-4[a]	0.97	s	-	-	X			
Uracil	5.80	d	1	=CH–	X	X	X	
Uridine	5.88	d	1	=CH–	X	X	X	
Valine	1.03	d	3	$–CH_3$	X	X	X	
Xylose	5.18	d	1	>CH–		X	X	X

[a] The unknown signals were included as Lorentzians to model signal interference in the root exudate spectra (see Fig. S1) but they were not quantitatively interpreted.

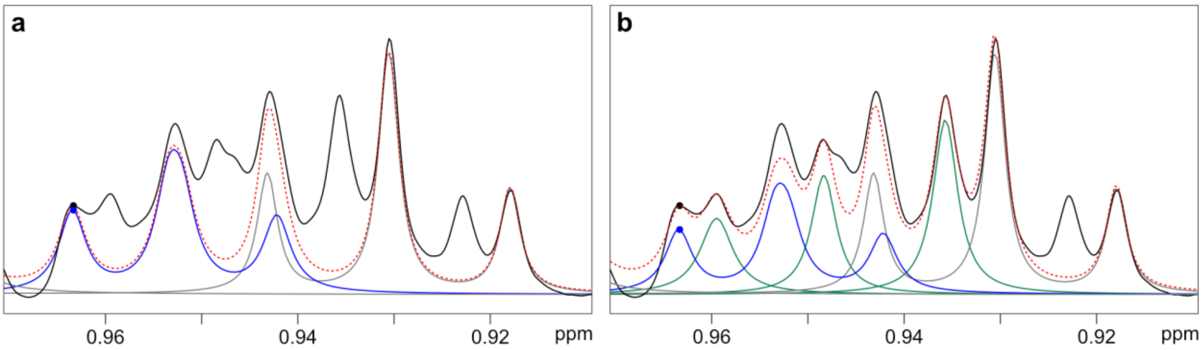

Fig. S1 Overlap with unknown signals. The leucine signal that was selected for AQuA (blue) overlaps with unknown signals that need to be included in the model for accurate concentration estimation. The red dashed line represents the model sum. When only known metabolites (blue and grey) are included in the model, the calculated height of the leucine signal (blue dot) closely matches that of the experimental target signal (black dot) (a). When unknown signals (green) are added to the model, the leucine signal is estimated to be less intense, because interference contributions from the unknown signals are now taken into account (b). The library signals of the unknowns were generated in Chenomx as Lorentzians whose positions and signal line widths were matched manually, based on visual inspection, to one representative root exudate NMR spectrum.

2. Evaluation of different methods for suppression of broad signals in 1D-^{1}H NMR spectra

2.1 Evaluation results

Ultrafiltration is the routine procedure to remove macromolecules from blood samples. However, the broad signals in the root exudate sample were unaffected by this procedure, meaning that they are likely not caused by macromolecules larger than 3 kDa (Fig. S2). The sample was also passed through a C18 solid phase extraction (SPE) column. In the aqueous filtrate, some of the broad signals have been completely removed whereas others have been diminished. The baseline was also generally improved. However, this procedure inevitably disturbs the integrity of the sample and some compounds, including the internal standard DSS, were not fully recovered in the first elution step but appear in the subsequent methanolic eluate as well (Fig. S3). Thus, the method cannot be used for absolute metabolite quantification. Furthermore, since some broad signals are still present in the aqueous filtrate, it can be concluded that the underlying compounds are not particularly hydrophobic.

The CPMG pulse sequence (Carr & Purcell 1954; Meiboom & Gill 1958) is a common approach to selectively suppress broad NMR signals based on their short transverse relaxation times (T_2) compared to narrower signals. Here, with the CPMG experiment it was possible to reduce the intensity of the broad signals, but not without severely affecting certain metabolite signals (Fig. S2). Diffusion experiments, in which resonances from fast-diffusing compounds are defocused, also allowed the intensity of the broad signals in the difference spectrum to be reduced, but other signals were suppressed as well (Fig. S2 and S4). Thus, it appears that there is no distinct difference in T_2 relaxation times or diffusion coefficients between the compounds causing the broad signals and the metabolites of interest, and it was therefore not possible to find NMR parameters that selectively targeted all broad signals, but no metabolite signals, using either CPMG or diffusion experiments. The diffusion experiments also affected individual broad signals differently, implying that the underlying compounds have different diffusion coefficients. This particular problem has been addressed in a previous study where the intensities of individual lipoprotein signals were allowed to vary ±10 % to achieve a good fit with the original 1D-^{1}H spectrum (de Graaf et al. 2015). However, because the spread in diffusion coefficients is much larger in our study, a higher intensity variation would have to be allowed to accurately fit the spectra.

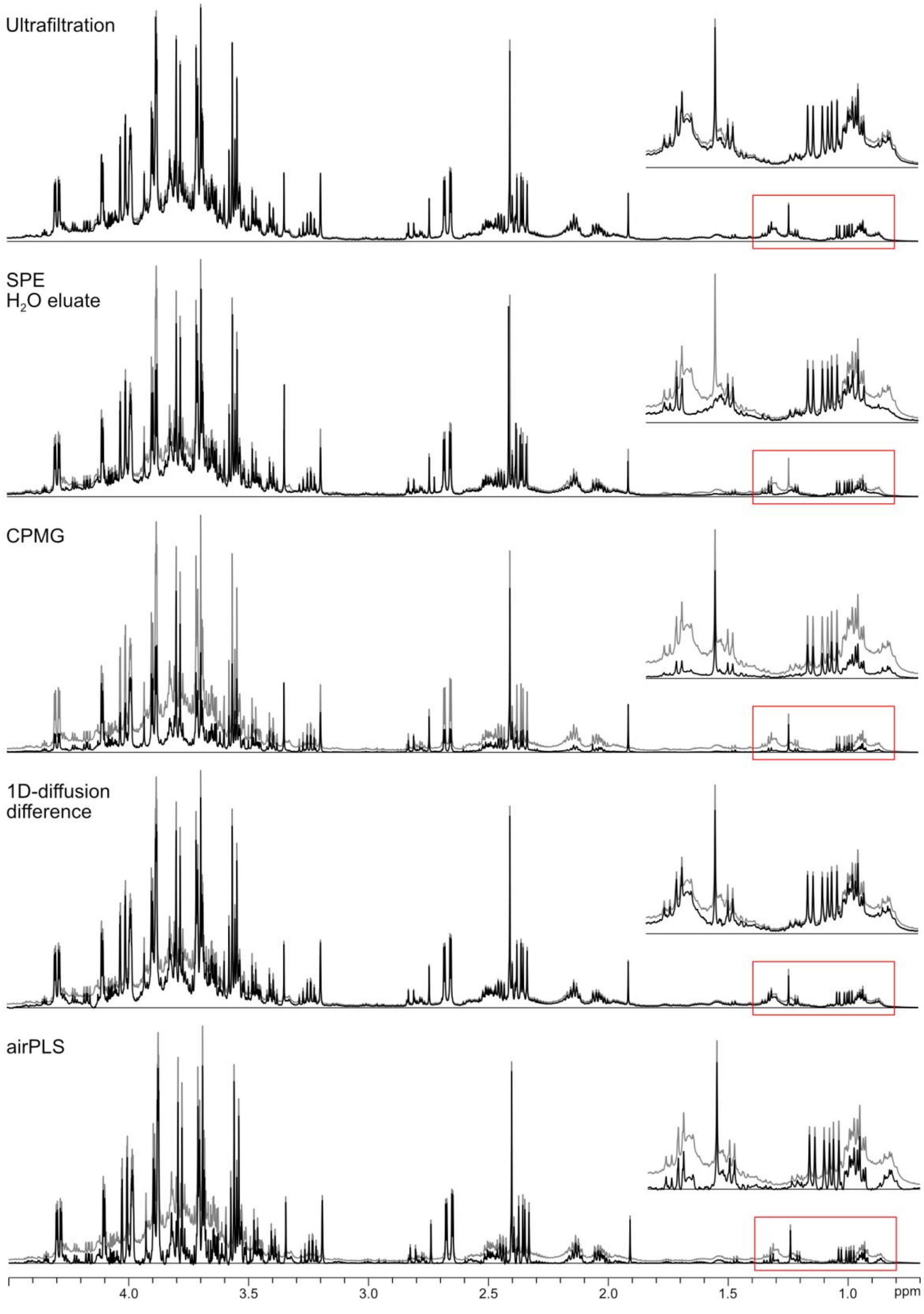

Fig. S2 Ultrafiltration, SPE, CPMG, 1D-diffusion, and airPLS applied to the same root exudate sample (black spectra). All NMR spectra are overlaid with a 1D-NOESY presaturation spectrum recorded on the intact sample (grey). A baseline at zero intensity has been added to all spectra. The insets show magnifications of the spectral region 0.8-1.4 ppm. For the airPLS computation, the default λ value was 1×10^7. In addition, two local λ values were used: 1×10^5 for the spectral region 1.23-1.33 ppm and 1×10^6 for the region 0.90-0.97 ppm. For the other methods, see section 2.2 for parameter details.

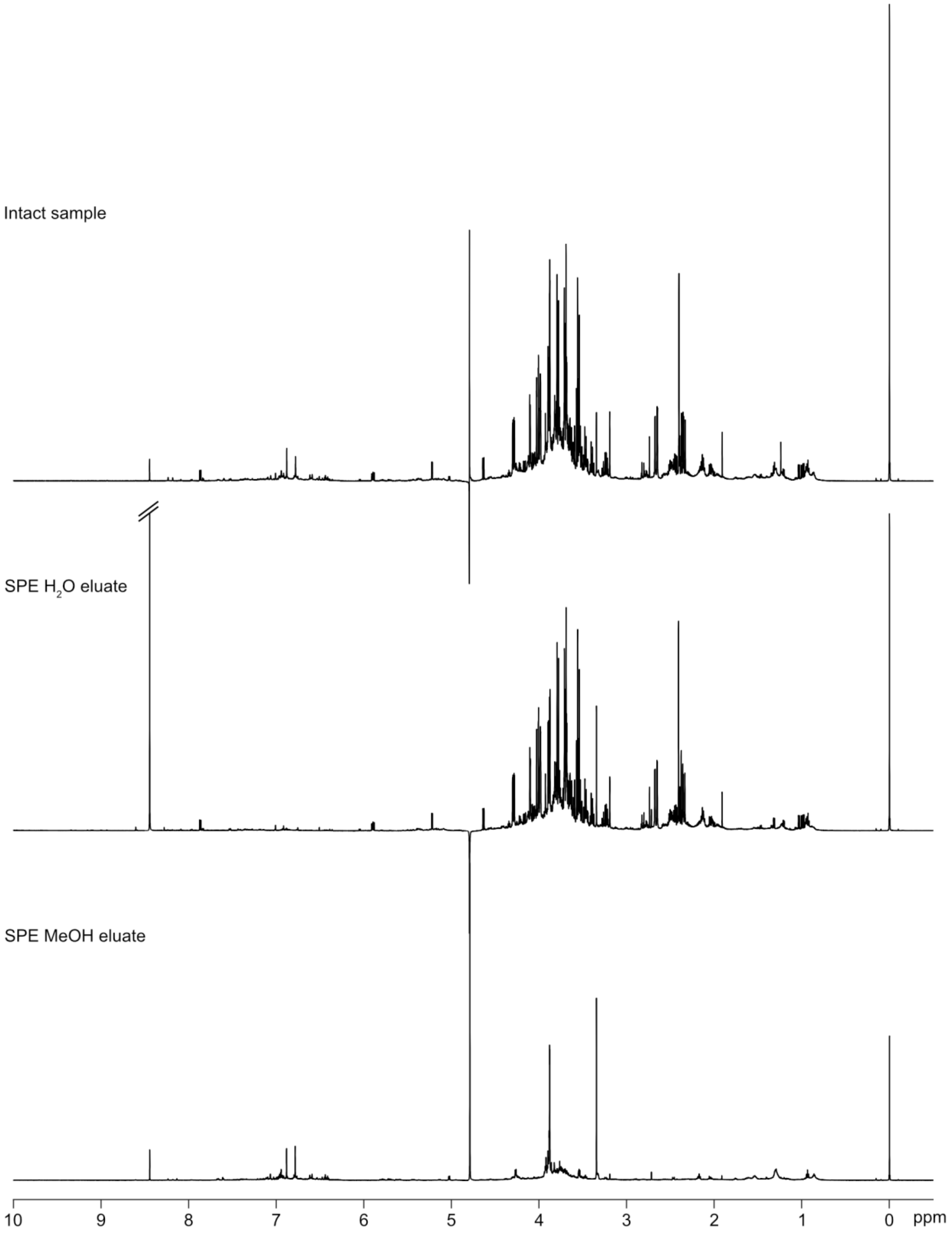

Fig. S3 1D-^{1}H NMR spectra of a pooled root exudate sample either analysed directly (top) or after it was passed through an SPE column (middle and bottom)

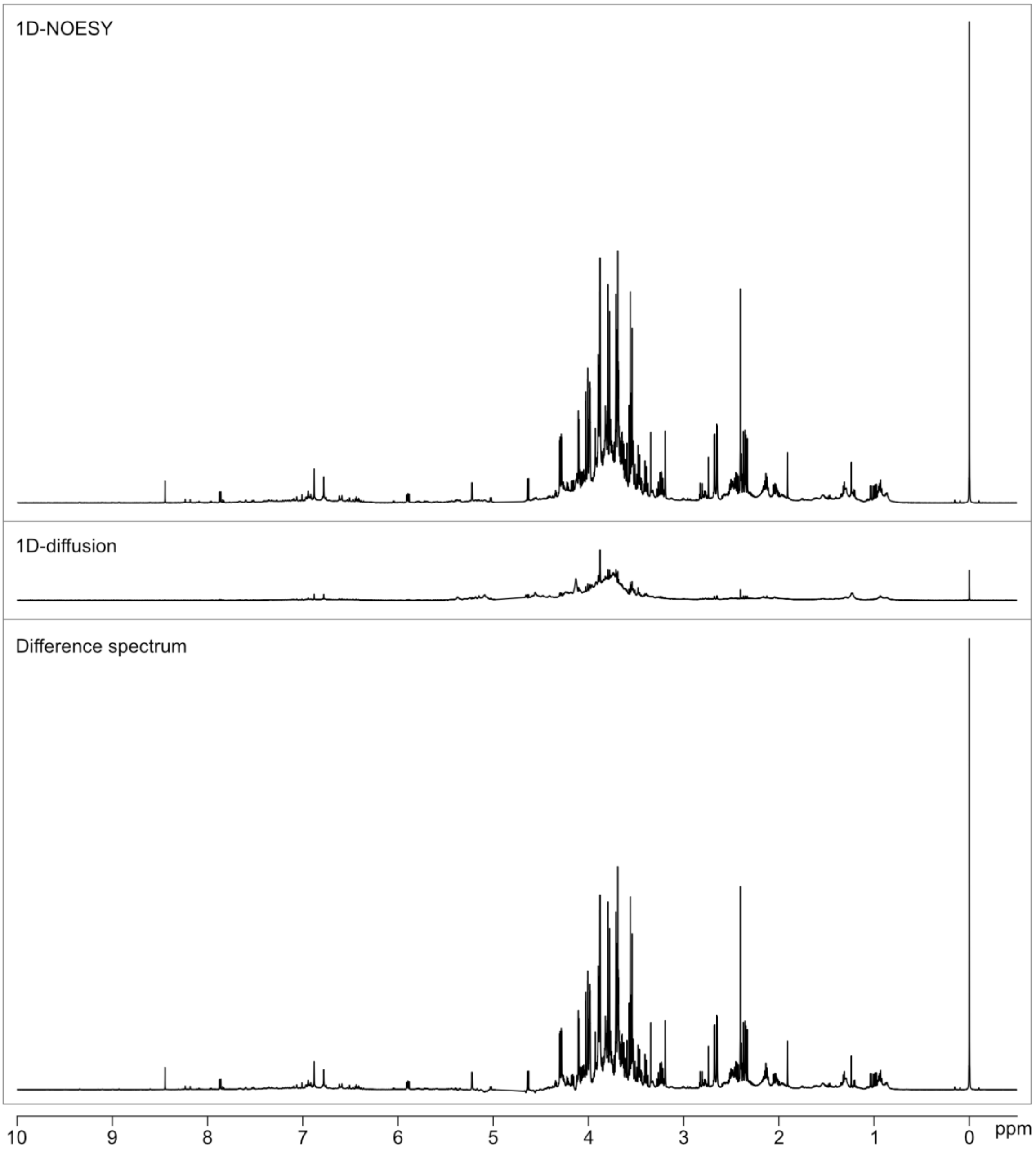

Fig. S4 1D-NOESY-presat, 1D-diffusion, and the difference spectrum (1D-diffusion subtracted from 1D-NOESY), respectively, of a pooled root exudate sample

2.2 Sample preparation and acquisition parameters

Ten of the analysed root exudate samples were pooled together to obtain a large enough sample volume for comparison of different methods. 600 µl of the pooled sample was transferred directly to an NMR tube to be used for 1D-NOESY-presat, CPMG, and diffusion-edited experiments. The remaining sample volume was used to evaluate ultrafiltration and solid phase extraction (SPE). Except where noted, NMR spectra were recorded and processed as described in the Materials and methods section of the paper.

Ultrafiltration was performed in two replicates using Nanosep filters with 3 kDa cut-off (Pall Life Science, Port Washington (NY), USA). The filters were washed nine times with MilliQ water (500 µl, 36 °C, 2000 *g*, 15 min) and then once with D_2O before the sample was added. To each filter, 500 µl of the pooled sample was added and the samples were spun for 10 min at 13 000 *g* and 4 °C, after which an additional volume of 200 µl was added and the centrifugation was repeated until most of the sample had passed through the filter. 600 µl of the filtered samples were then transferred to NMR tubes.

SPE was performed in two replicates using Isolute C18(EC) cartridges (50 mg, 1 ml) (Biotage, Uppsala, Sweden). The cartridges were activated with 1 ml methanol and then washed with 1 ml MilliQ water. Thereafter, 600 µl of the pooled sample was passed through each column followed by 1 ml MilliQ water to wash out all polar metabolites. To elute non-polar metabolites from the SPE columns, 1 ml methanol was added and the eluate was collected separately. Both the aqueous (sample and subsequent MilliQ washing) and the methanolic eluates were dried in a vacuum centrifuge. The dried samples were dissolved in 600 µl D_2O and transferred to NMR tubes.

CPMG spectra were recorded with water presaturation using the Bruker pulse sequence *cpmgpr1d* (relaxation delay-90°-$(\tau\text{-}180°\text{-}\tau)_n$-acquire). The best suppression of broad signals was achieved with a 2 s relaxation delay, n = 450 loops, and a spin echo delay τ of 1 ms, yielding a total echo time of 900 ms.

Diffusion-edited spectra were recorded using a longitudinal-eddy current delay experiment with bipolar gradients (the Bruker pulse sequence *ledbpgp2s1d*). The best suppression of broad signals in the difference spectrum was achieved with a diffusion time of 100 ms and an effective gradient pulse duration (δ) of 2 ms. The gradient strength was set to 95 % of its maximum value (the maximum z-gradient was 48.15 G/cm). 1024 transients were recorded in the diffusion experiment to improve the signal to noise ratio.

3. Simulated spectra

The spectral background of one root exudate spectrum was modelled using average-based smoothing. The procedure is outlined in Fig. S5a. Before applying the smoothing function, narrow high-intensity signals ("spikes") were removed from the spectrum. This was done by defining spike borders - two spectral data points on either side of the spike - and performing linear regression between these two points. Smoothing applied directly to the root exudate spectrum resulted in undesirable artefacts in spike regions (Fig. S5b), which is why spikes were removed before the smoothing step.

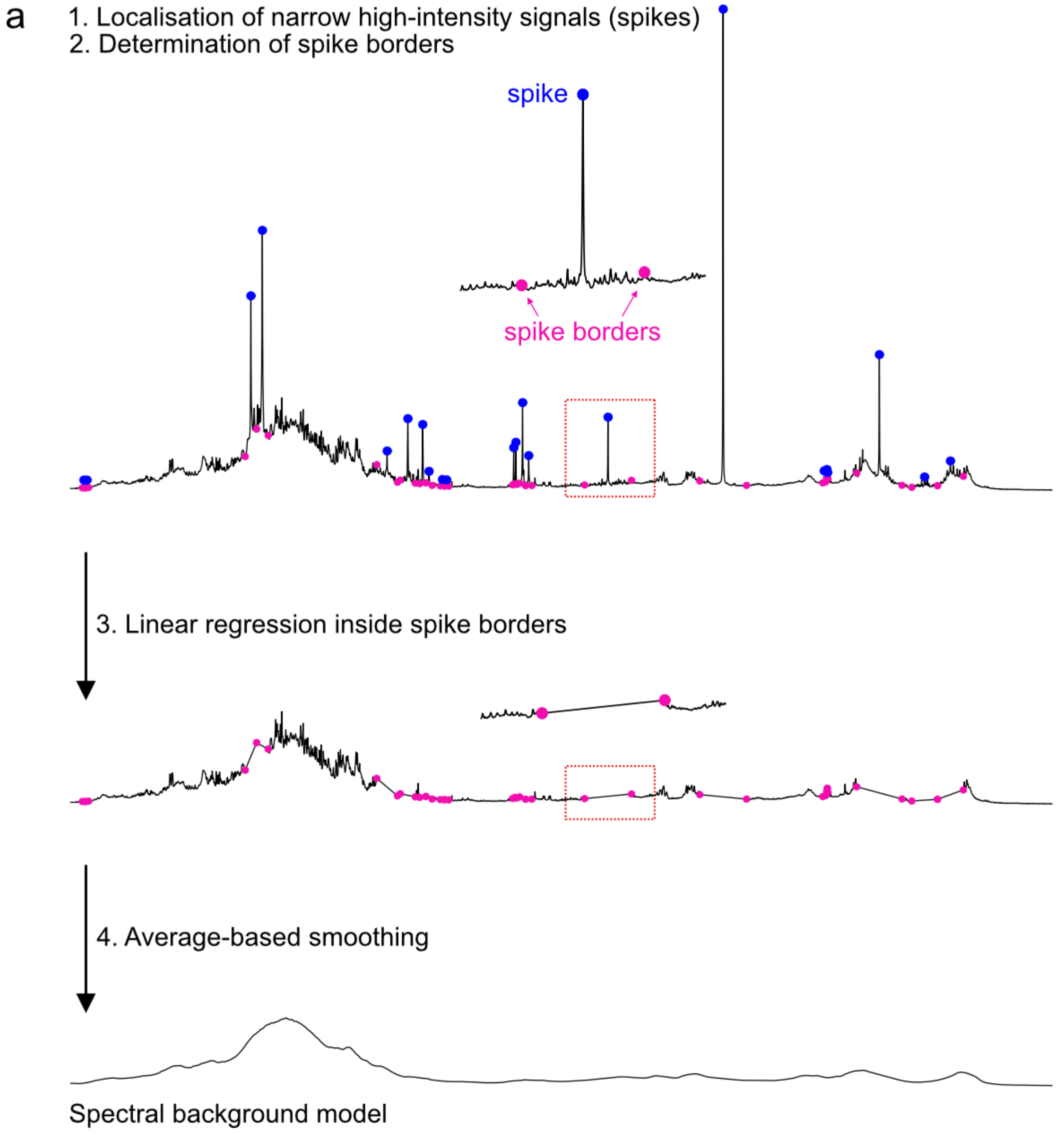

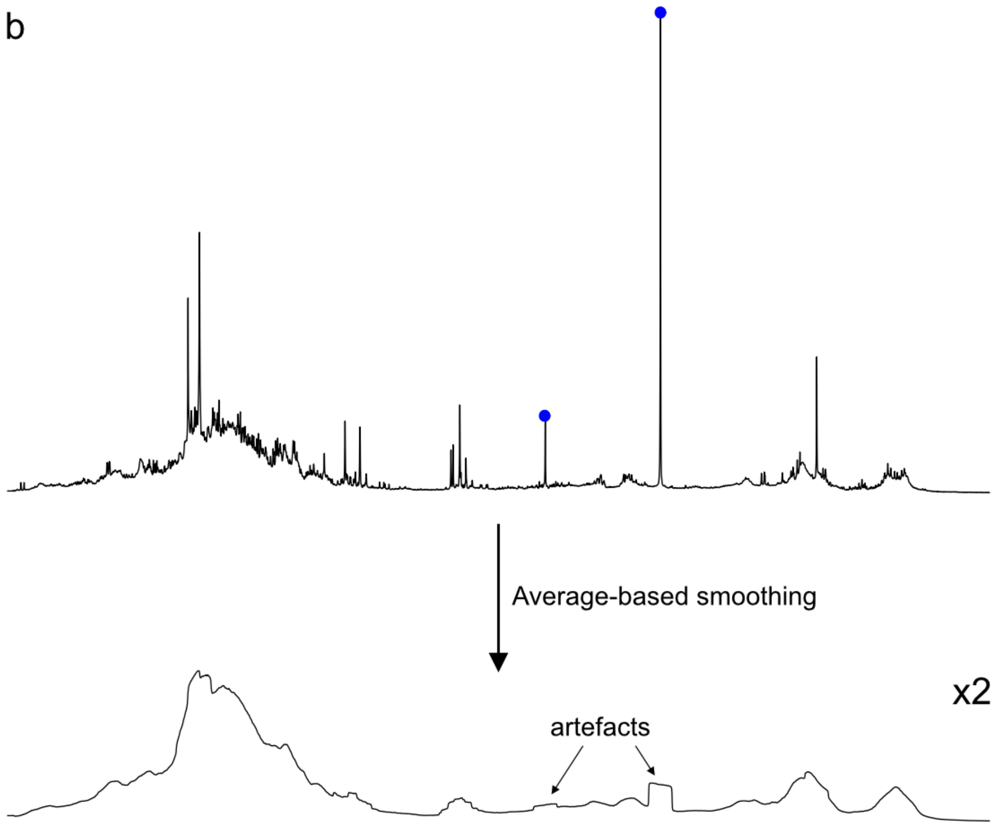

Fig. S5 Modelling the spectral background of a root exudate spectrum. a) The procedure used here to generate the spectral background models (background B is shown), b) Average-based smoothing applied to the root-exudate spectrum with spikes creates artefacts in spike regions (indicated here for two of the spikes).

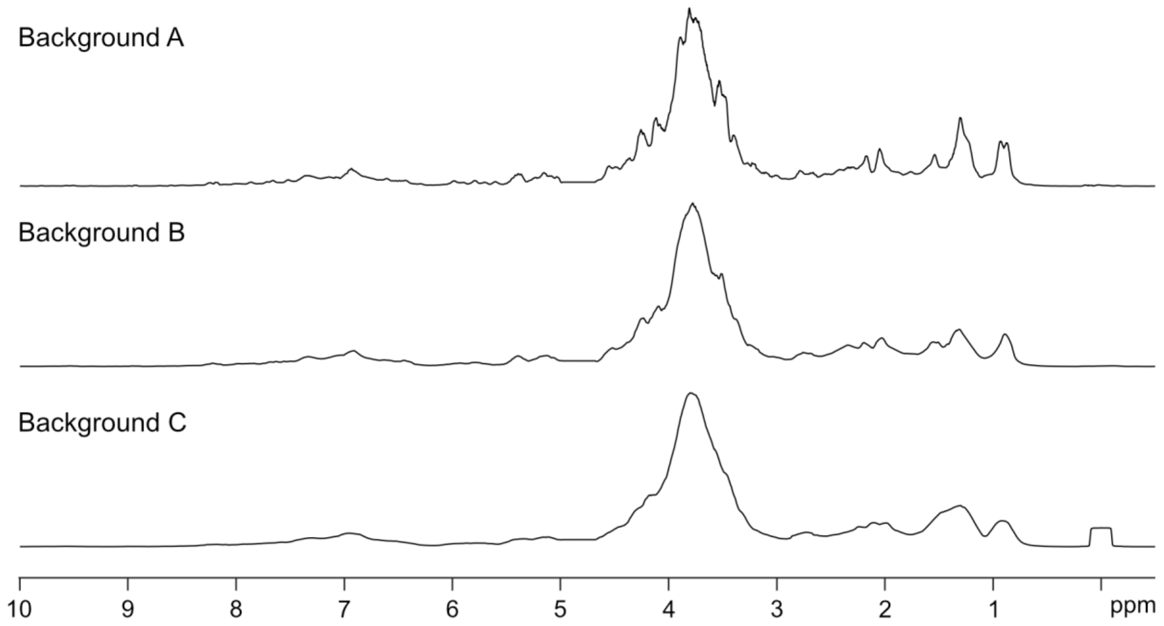

Fig. S6 The three spectral background models included in the simulations

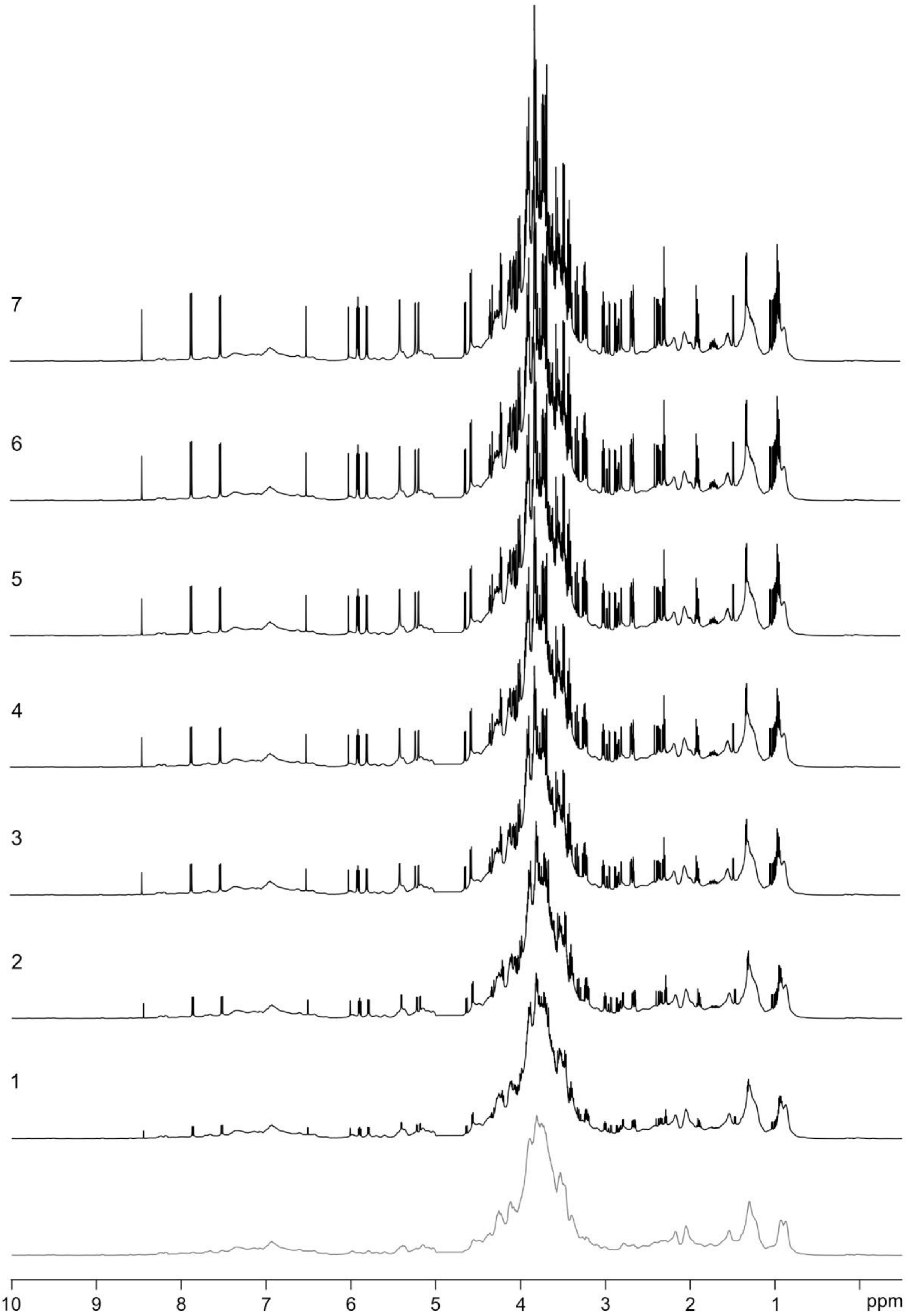

Fig. S7 The seven simulated spectra (black) based on background A (grey)

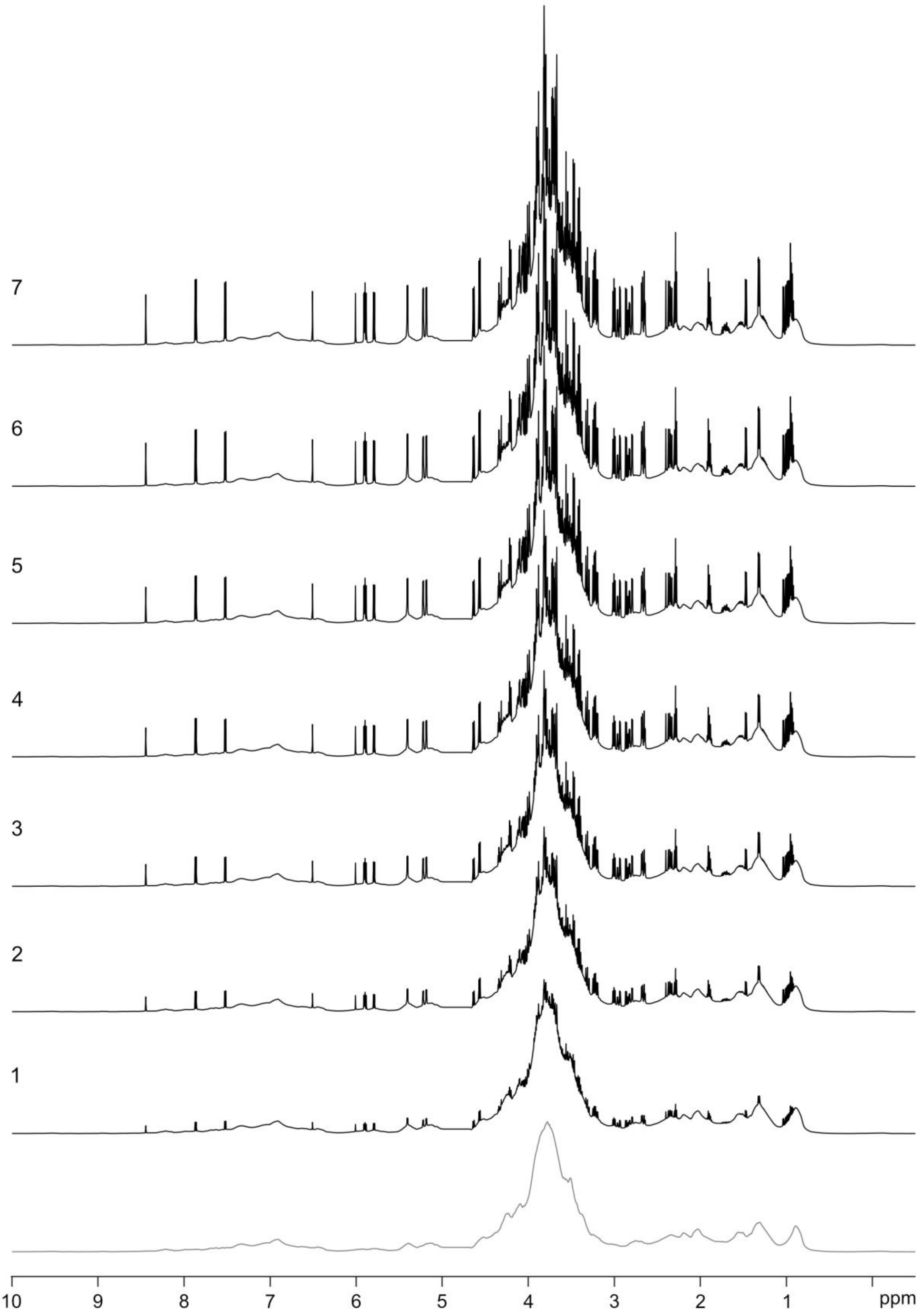

Fig. S8 The seven simulated spectra (black) based on background B (grey)

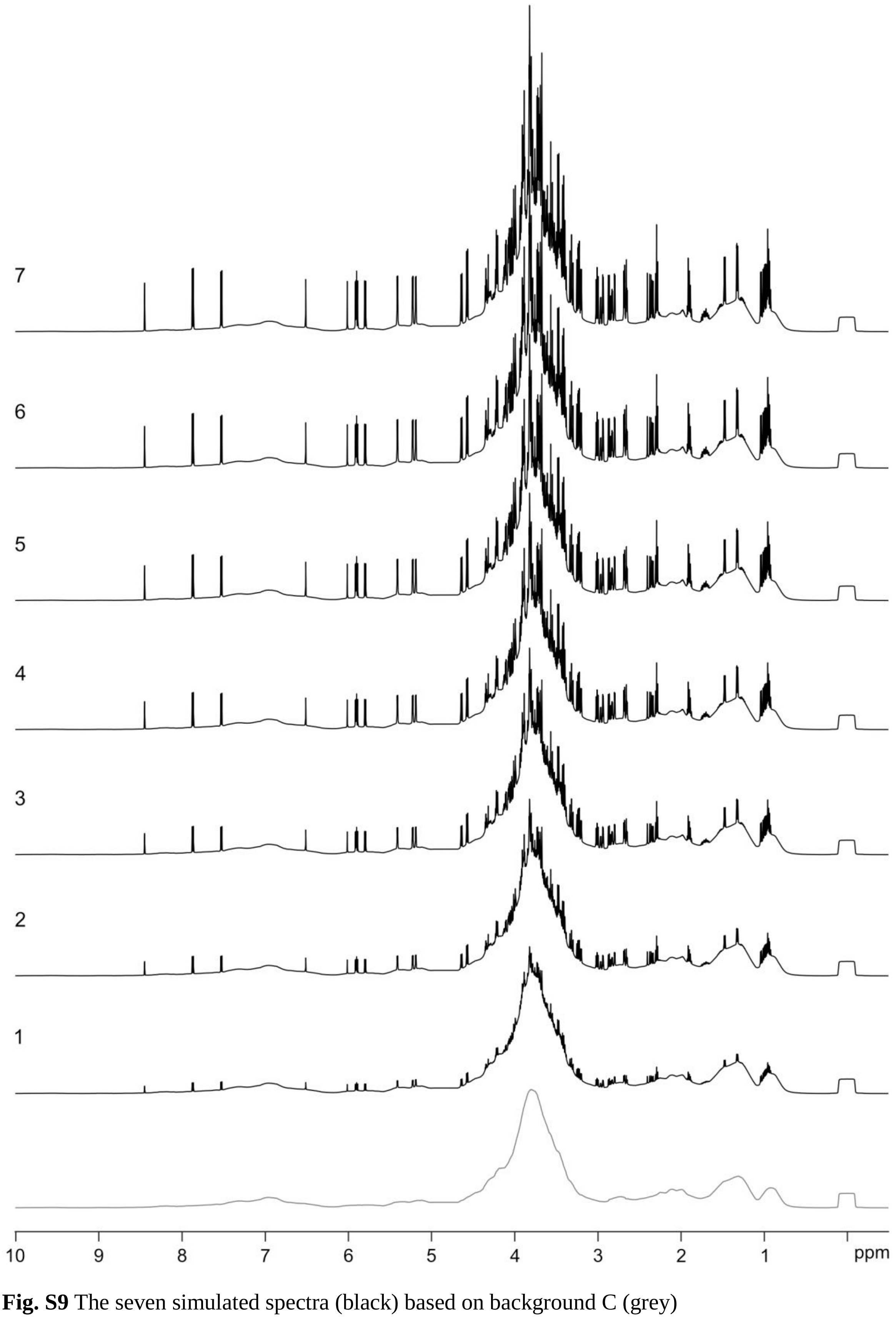

Fig. S9 The seven simulated spectra (black) based on background C (grey)

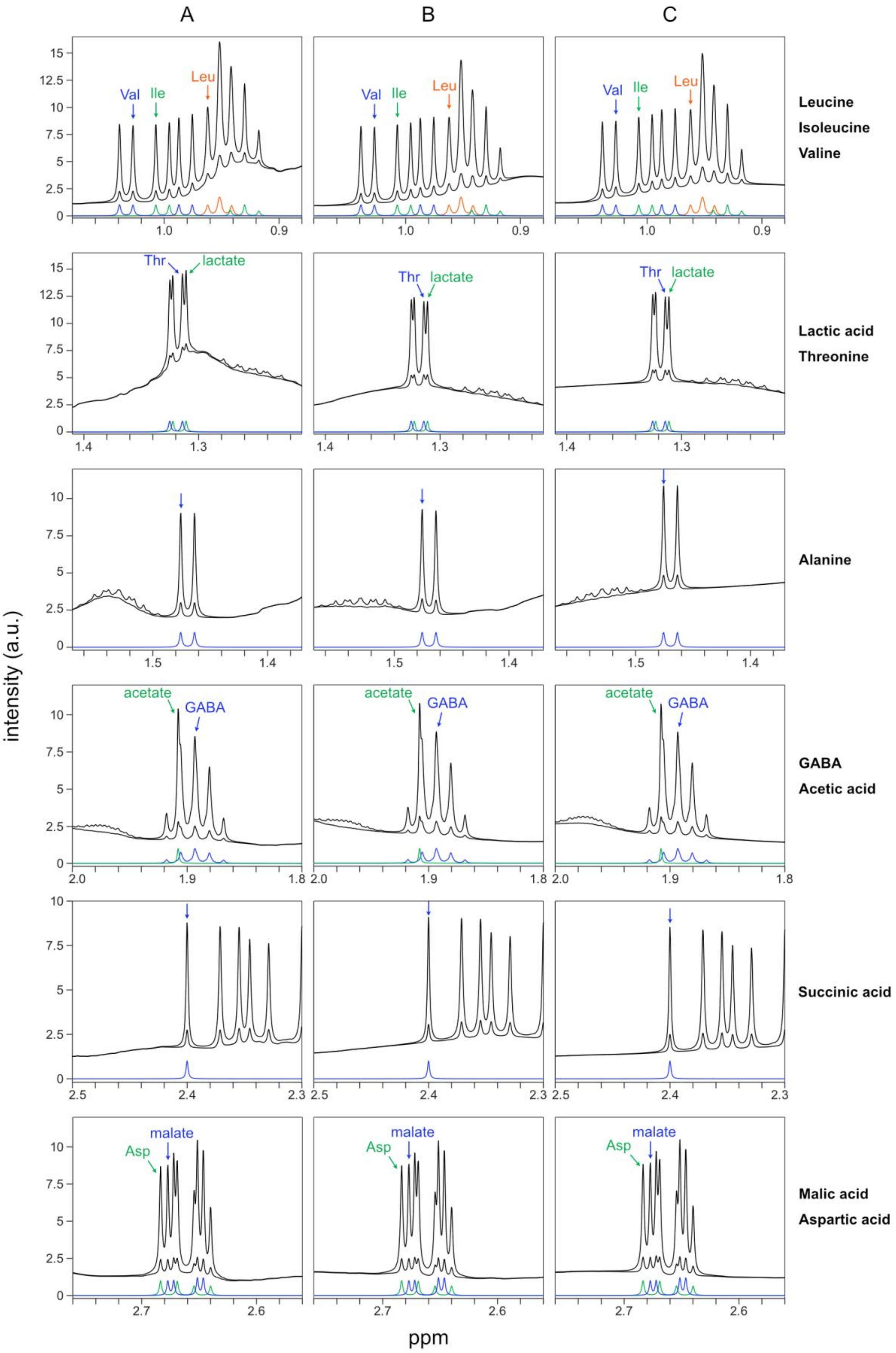
A
B
C
Val
Ile
Leu
Leucine
Isoleucine
Valine
Thr
lactate
Lactic acid
Threonine
Alanine
acetate
GABA
GABA
Acetic acid
Succinic acid
Asp
malate
Malic acid
Aspartic acid
intensity (a.u.)
ppm

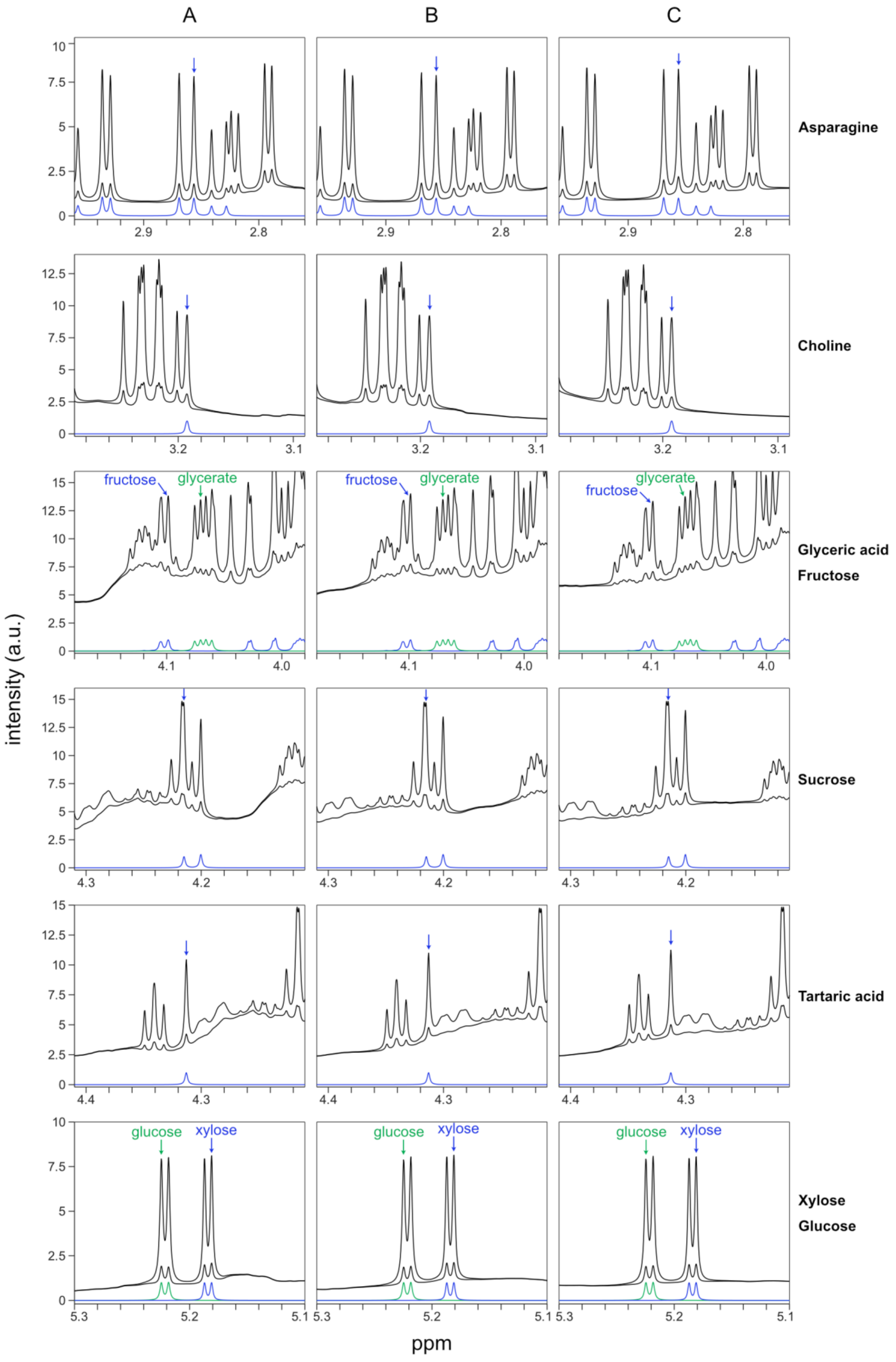
A
B
C
Asparagine
Choline
fructose
glycerate
Glyceric acid
Fructose
Sucrose
Tartaric acid
glucose
xylose
Xylose
Glucose
intensity (a.u.)
ppm

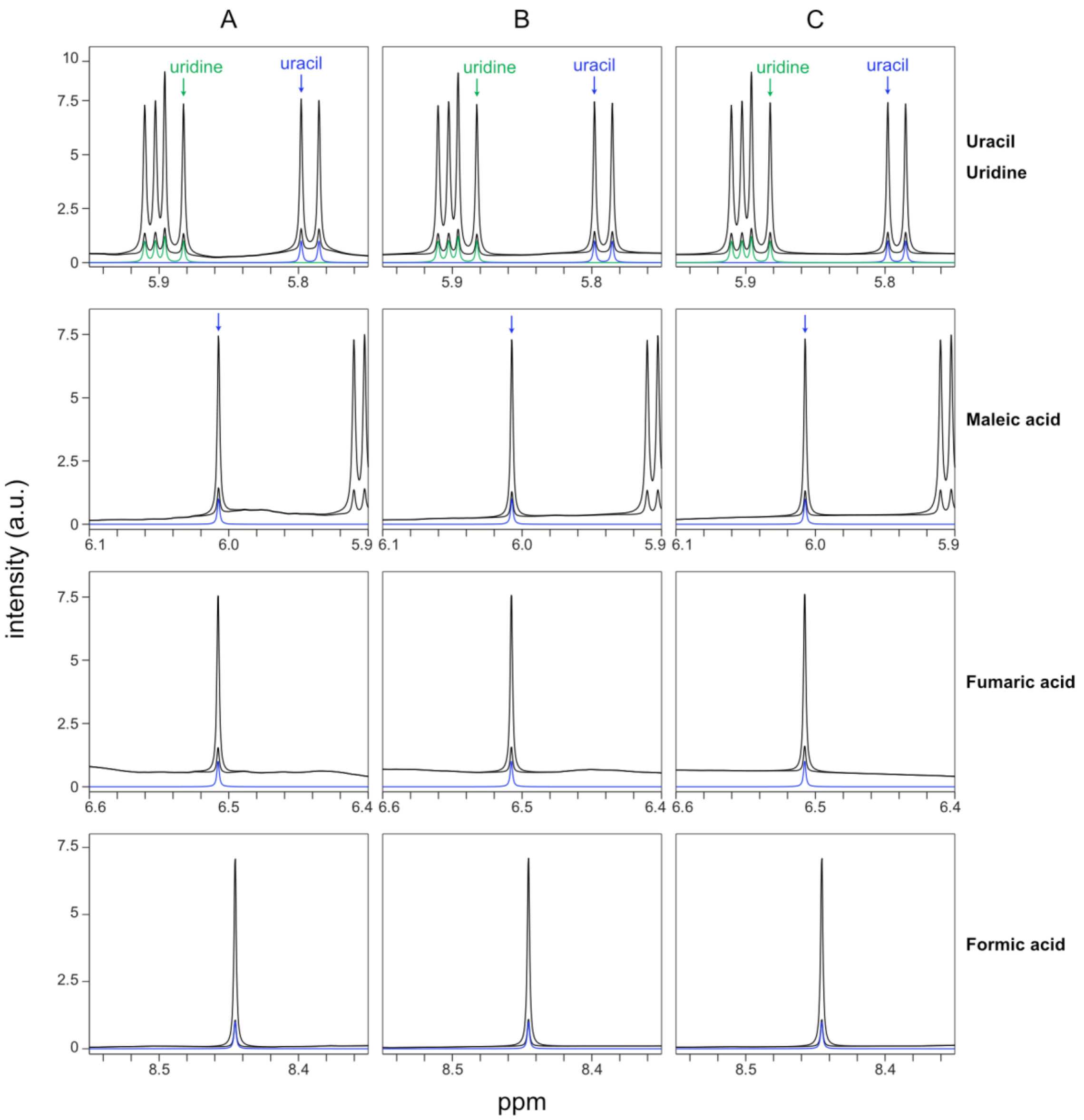

Fig. S10 The metabolite signals that were targeted in the analysis of the simulated spectra. Shown are the uncorrected simulated spectra with the lowest and highest proportion of narrow signal to spectral background for background A, B, and C (i.e. spectra 1 and 7 in Fig. S6-S8) (black). Shown are also the corresponding library spectra (coloured), normalised so that the intensities of the signals used in AQuA equal 1 a.u.

Table S2 The contribution (in percent) of the spectral background to the total target signal intensities in the simulated root exudate NMR spectra[a]

	Spectrum 1			Spectrum 7		
	**A**	**B**	**C**	**A**	**B**	**C**
Acetic acid	55.0	60.1	59.6	14.9	17.7	17.4
Alanine	66.8	69.3	79.3	22.3	24.4	35.4
Asparagine	44.3	45.9	54.5	10.2	10.8	14.6
Aspartic acid	57.3	58.2	59.7	16.1	16.6	17.5
Choline	67.0	66.0	64.3	22.3	21.7	20.5
Formic acid	6.3	8.7	7.8	1.0	1.3	1.2
Fructose	86.2	86.6	85.3	47.2	47.9	45.3
Fumaric acid	35.2	36.2	37.7	7.2	7.5	8.0
GABA	60.1	64.4	64.3	17.7	20.5	20.5
Glucose	47.8	46.3	47.5	11.6	11.0	11.4
Glyceric acid	86.0	86.0	86.5	46.7	46.7	47.8
Isoleucine	56.1	55.9	66.1	15.4	15.3	21.8
Lactic acid	86.2	78.7	80.3	47.1	34.5	36.8
Leucine	74.0	65.7	72.3	28.8	21.5	27.2
Maleic acid	30.6	22.4	24.4	5.9	4.0	4.4
Malic acid	56.5	57.5	58.9	15.6	16.2	17.0
Succinic acid	63.5	67.2	59.9	19.9	22.6	17.6
Sucrose	79.3	79.1	79.4	35.4	35.0	35.4
Tartaric acid	75.5	78.3	79.4	30.6	34.1	35.5
Threonine	85.6	78.7	80.3	46.0	34.5	36.8
Uracil	36.6	30.7	29.0	7.6	6.0	5.5
Uridine	25.7	24.2	28.5	4.7	4.4	5.4
Valine	55.7	52.5	62.3	15.2	13.6	19.1
Xylose	52.1	52.8	50.7	13.5	13.8	12.8

[a] Estimated by comparing the signal intensities in the simulated root exudate spectra, before correction with the airPLS algorithm, to the intensities in the corresponding narrow signal spectra. Included in the table are estimates for the simulated spectra with the lowest (1) and highest (7) proportion of narrow signals to spectral background, for each background (A, B, and C).

Table S3 Evaluation of the extended AQuA applied to 21 simulated NMR spectra[a]

	λ	A				B				C			
		**slope**	**intercept**	**R^2**	**diff %[b]**	**slope**	**intercept**	**R^2**	**diff %[b]**	**slope**	**intercept**	**R^2**	**diff %[b]**
Acetic acid	10^6	0.9370	-0.0931	0.9997	8.7	0.9017	-0.0087	1.0000	10.1	0.9013	-0.0162	1.0000	10.4
	10^7	0.9885	-0.0108	1.0000	1.5	0.9864	-0.0787	0.9998	3.6	0.9847	-0.1254	0.9995	4.8
	10^8	0.9929	0.0316	1.0000	0.5	0.9926	-0.0103	1.0000	1.0	0.9898	-0.0739	1.0000	3.1
Alanine	10^6	0.9856	-0.0028	0.9999	1.5	0.9686	-0.0560	1.0000	5.2	0.9709	-0.0280	1.0000	4.0
	10^7	0.9864	0.0500	1.0000	1.0	0.9800	-0.0718	1.0000	4.6	0.9872	0.0109	1.0000	1.1
	10^8	1.0029	0.4138	0.9996	15.3	1.0036	0.1328	0.9999	5.3	0.9962	0.0829	0.9995	3.1
Asparagine	10^6	0.9534	-0.0233	1.0000	5.4	0.9452	-0.0287	1.0000	6.6	0.9457	0.0196	1.0000	4.9
	10^7	0.9562	-0.0110	1.0000	4.8	0.9580	-0.0531	1.0000	6.1	0.9802	0.0146	0.9991	2.3
	10^8	0.9876	-0.0558	1.0000	3.3	0.9865	-0.0851	1.0000	4.4	0.9913	0.0974	0.9997	3.1
Aspartic acid	10^6	0.9548	-0.0584	0.9999	6.4	0.9454	-0.0097	1.0000	5.9	0.9443	0.0206	1.0000	5.0
	10^7	0.9787	0.1066	0.9999	1.9	0.9848	-0.0460	0.9995	2.8	0.9750	-0.0085	0.9997	2.5
	10^8	0.9913	0.2224	1.0000	7.0	0.9939	0.0690	1.0000	1.9	0.9876	0.0223	0.9999	1.5
Choline	10^6	0.9322	0.0167	1.0000	6.1	0.9393	-0.0458	1.0000	7.8	0.9389	-0.0204	1.0000	6.9
	10^7	0.9560	0.1190	1.0000	2.5	0.9781	-0.1462	0.9990	6.9	0.9568	-0.0531	0.9996	5.9
	10^8	0.9625	0.4813	0.9992	13.4	0.9935	0.2393	1.0000	8.0	0.9844	-0.0539	0.9999	3.3
Formic acid	10^6	0.9952	-0.0083	1.0000	0.8	0.9917	-0.0037	1.0000	1.0	0.9916	-0.0041	1.0000	1.0
	10^7	0.9978	-0.0028	1.0000	0.3	0.9985	-0.0036	1.0000	0.3	0.9981	-0.0033	1.0000	0.3
	10^8	0.9987	0.0095	1.0000	0.2	0.9992	0.0053	1.0000	0.1	0.9991	0.0027	1.0000	0.1
Fructose	10^6	0.9105	-0.1803	0.9931	14.6	0.8485	0.0302	0.9999	14.3	0.8471	-0.0124	0.9999	15.9
	10^7	0.8125	0.9849	0.9583	20.1	0.9362	0.0799	0.9998	3.7	0.9298	-0.0789	1.0000	9.8
	10^8	0.9223	1.7648	0.9998	55.0	0.9703	1.0166	0.9963	34.3	0.9376	-0.1367	0.9999	10.9
Fumaric acid	10^6	0.9906	-0.0264	1.0000	1.8	0.9908	-0.0058	1.0000	1.2	0.9910	-0.0023	1.0000	1.0
	10^7	0.9980	-0.0203	1.0000	0.9	0.9971	0.0018	1.0000	0.2	0.9977	0.0081	1.0000	0.2
	10^8	0.9979	0.0234	1.0000	0.6	1.0055	-0.0121	1.0000	0.4	0.9992	0.0189	1.0000	0.6
GABA	10^6	0.9128	-0.1571	0.9985	13.8	0.8431	-0.0094	1.0000	16.1	0.8442	-0.0060	1.0000	15.9
	10^7	0.9844	-0.0242	0.9999	2.4	0.9849	-0.1151	0.9992	5.6	0.9805	-0.1246	0.9987	5.9
	10^8	0.9919	0.0617	1.0000	1.5	0.9909	-0.0045	1.0000	1.0	0.9884	-0.0481	1.0000	2.9
Glucose	10^6	0.9798	-0.0242	0.9998	2.6	0.9679	-0.0374	1.0000	4.7	0.9674	-0.0128	1.0000	3.8
	10^7	0.9824	0.0689	1.0000	1.3	0.9829	-0.0465	1.0000	3.4	0.9828	-0.0056	1.0000	1.9
	10^8	0.9986	0.2701	0.9839	9.0	0.9868	0.0194	0.9988	2.1	0.9929	-0.0047	0.9999	1.0
Glyceric acid	10^6	0.8906	-0.0922	0.9997	14.0	0.8795	-0.2530	1.0000	21.2	0.8871	-0.0915	0.9999	14.8
	10^7	0.8247	0.4699	0.9815	14.7	0.8936	-0.3004	0.9998	21.3	0.9065	-0.0224	0.9994	9.7
	10^8	0.9014	1.1563	0.9997	32.0	0.9565	0.3315	0.9978	8.5	0.9174	-0.0180	0.9997	8.6
Isoleucine	10^6	0.9373	-0.0237	0.9998	6.9	0.9204	-0.0064	1.0000	8.2	0.9195	-0.0137	1.0000	8.6
	10^7	0.9225	-0.0142	1.0000	8.1	0.9355	-0.0218	1.0000	7.2	0.9333	-0.0265	1.0000	7.6
	10^8	0.9403	-0.0888	0.9985	9.2	0.9373	-0.0729	1.0000	8.9	0.9536	-0.0051	0.9991	4.4
Lactic acid	10^6	0.9017	0.7359	0.9864	16.8	0.9597	-0.0227	1.0000	4.9	0.9566	-0.0468	1.0000	6.0
	10^7	0.9918	1.4528	0.9983	47.4	0.9950	0.0414	0.9997	1.4	0.9860	-0.0060	0.9999	1.5
	10^8	0.9452	3.5842	0.9950	112.4	1.0034	0.5829	0.9993	20.0	0.9968	0.0978	0.9964	4.4
Leucine	10^6	0.8941	-0.2236	0.9973	17.8	0.8520	-0.0403	0.9998	16.6	0.8528	-0.0446	0.9999	16.6
	10^7	0.8877	0.2504	0.9998	6.2	0.8817	-0.1065	0.9998	15.5	0.8810	-0.0367	0.9997	13.0
	10^8	0.9171	1.1772	0.9815	34.1	0.8833	0.0747	0.9981	8.8	0.9583	-0.0278	0.9962	5.3
Maleic acid	10^6	0.9871	0.0310	1.0000	1.1	0.9894	-0.0013	1.0000	1.1	0.9890	-0.0062	1.0000	1.4
	10^7	1.0003	0.0997	0.9999	3.5	0.9969	-0.0017	1.0000	0.4	0.9972	-0.0091	1.0000	0.6
	10^8	0.9906	0.2288	0.9998	7.5	0.9985	0.0040	1.0000	0.1	1.0004	-0.0011	1.0000	0.1

Table S3 (continued) Evaluation of the extended AQuA applied to 21 simulated spectra[a]

		A				B				C			
	λ	slope	intercept	R^2	diff %[b]	slope	intercept	R^2	diff %[b]	slope	intercept	R^2	diff %[b]
Malic acid	10^6	0.9422	-0.1181	0.9999	9.7	0.9326	0.0038	1.0000	6.8	0.9310	0.0402	0.9999	5.7
	10^7	0.9772	0.0961	0.9999	1.6	0.9840	-0.0629	0.9991	3.4	0.9725	-0.0139	0.9994	2.9
	10^8	0.9912	0.2170	1.0000	6.7	0.9939	0.0745	1.0000	2.0	0.9880	0.0168	0.9999	1.5
Succinic acid	10^6	0.9941	-0.0044	0.9999	0.8	0.9885	-0.0041	1.0000	1.3	0.9888	-0.0041	1.0000	1.3
	10^7	0.9833	0.1030	1.0000	2.2	0.9869	-0.0021	1.0000	1.4	0.9866	0.0024	1.0000	1.3
	10^8	0.9833	0.1013	1.0000	2.1	0.9890	-0.0066	1.0000	1.3	0.9893	-0.0041	1.0000	1.2
Sucrose	10^6	0.9329	-0.0593	0.9967	8.5	0.8868	-0.0314	1.0000	12.3	0.8823	0.1062	0.9992	9.5
	10^7	0.9010	0.9155	0.9810	14.6	0.9910	-0.0534	0.9997	2.2	0.9619	0.1748	0.9996	3.2
	10^8	0.9887	1.2913	0.9999	33.7	0.9796	0.5537	0.9998	12.8	0.9709	0.2819	1.0000	4.8
Tartaric acid	10^6	0.9618	-0.0768	0.9983	6.2	0.9183	0.0690	1.0000	5.8	0.9249	0.0055	1.0000	7.4
	10^7	0.9508	0.0241	0.9982	4.6	0.9567	0.0345	1.0000	3.1	0.9632	0.0305	0.9999	2.8
	10^8	0.9575	0.2397	1.0000	4.7	0.9603	0.2986	0.9993	6.9	0.9779	0.2955	0.9996	8.9
Threonine	10^6	0.9100	0.5333	0.9891	13.0	0.9577	-0.0385	1.0000	5.7	0.9544	-0.0539	0.9999	6.5
	10^7	0.9930	1.2197	0.9984	39.8	0.9947	0.0219	0.9997	1.0	0.9861	-0.0118	0.9999	1.6
	10^8	0.9455	3.2740	0.9951	102.1	1.0035	0.5905	0.9993	20.2	0.9968	0.0827	0.9970	3.9
Uracil	10^6	0.9877	0.0102	0.9999	1.4	0.9669	-0.0115	1.0000	3.8	0.9673	-0.0062	1.0000	3.6
	10^7	0.9972	0.1559	0.9999	5.5	0.9949	-0.0209	1.0000	1.3	0.9947	-0.0006	1.0000	0.5
	10^8	0.9964	0.3030	1.0000	10.8	0.9980	0.0773	1.0000	2.7	0.9979	0.0077	1.0000	0.2
Uridine	10^6	0.9562	-0.0183	1.0000	5.0	0.9493	-0.0047	1.0000	5.3	0.9493	-0.0005	1.0000	5.1
	10^7	0.9932	0.0556	0.9999	1.4	0.9905	-0.0403	1.0000	2.4	0.9889	-0.0227	0.9999	1.8
	10^8	0.9904	0.1235	0.9999	3.6	0.9949	-0.0142	1.0000	1.0	0.9958	0.0082	1.0000	0.4
Valine	10^6	0.9671	-0.0035	1.0000	3.4	0.9614	-0.0210	1.0000	4.8	0.9618	-0.0052	1.0000	4.2
	10^7	0.9593	0.1596	1.0000	3.2	0.9676	-0.0187	1.0000	3.9	0.9650	0.0211	1.0000	2.7
	10^8	0.9527	0.0594	0.9999	3.3	0.9609	-0.0337	1.0000	5.1	0.9633	0.0236	0.9999	2.9
Xylose	10^6	0.9764	-0.0113	1.0000	2.7	0.9737	-0.0153	1.0000	3.3	0.9744	-0.0094	1.0000	3.0
	10^7	0.9777	0.1173	1.0000	2.3	0.9855	-0.0032	1.0000	1.6	0.9838	-0.0047	1.0000	1.8
	10^8	0.9873	0.4527	0.9668	14.4	0.9818	0.1459	0.9938	3.8	0.9894	0.0209	0.9998	1.3
**Mean[c]**	**10^6**	**0.9496**	**0.1055**	**0.9982**	**7.0**	**0.9366**	**0.0316**	**1.0000**	**7.2**	**0.9367**	**0.0240**	**0.9999**	**6.8**
	**10^7**	**0.9584**	**0.2722**	**0.9964**	**8.0**	**0.9726**	**0.0572**	**0.9998**	**4.3**	**0.9700**	**0.0340**	**0.9998**	**3.6**
	**10^8**	**0.9723**	**0.6513**	**0.9966**	**20.1**	**0.9818**	**0.1856**	**0.9993**	**6.7**	**0.9818**	**0.0597**	**0.9994**	**3.2**
**Median[c]**	**10^6**	**0.9541**	**0.0287**	**0.9999**	**6.2**	**0.9473**	**0.0181**	**1.0000**	**5.7**	**0.9475**	**0.0133**	**1.0000**	**5.4**
	**10^7**	**0.9806**	**0.1014**	**0.9999**	**2.8**	**0.9848**	**0.0437**	**1.0000**	**3.3**	**0.9817**	**0.0143**	**0.9999**	**2.4**
	**10^8**	**0.9882**	**0.2342**	**0.9999**	**8.3**	**0.9918**	**0.0746**	**1.0000**	**4.1**	**0.9894**	**0.0230**	**0.9999**	**3.0**

[a] Results from linear regression including seven different intensity scaling levels (1-7 arbitrary intensity units) of the simulated narrow signal spectrum (summed library spectra). The three spectral background models were evaluated separately. Predictor (x-axis): Signal heights in the simulated narrow signal spectra (1, 2, 3, 4, 5, 6, and 7 arbitrary intensity units). Response (y-axis): Signal heights in the simulated spectra with both broad and narrow signals, after correction with the airPLS algorithm. Three λ values (1×10^6, 1×10^7, and 1×10^8) were evaluated.

[b] Average difference (%) between the signal intensities in the simulated narrow signal spectrum and the signal heights in the corresponding simulated spectra with both broad and narrow signals, after correction with the airPLS algorithm. The formula used was $100 \times |\text{Intensity}_{\text{narrow spectrum}} - \text{Intensity}_{\text{simulated spectrum}}| / \text{Intensity}_{\text{narrow spectrum}}$

[c] Mean and median were calculated on the absolute values of the intercepts

4. Spike-in experiment

4.1 Methodology

A spike-in experiment was performed to verify the linearity and accuracy of the method. The metabolites were chosen to represent different chemical classes and their selected target signals have varying coupling patterns and appear in different spectral regions, with or without interference from background or other metabolites (Table S4 and Fig. S10). None of the metabolites were present in the pooled sample before the spike-in experiment, as evaluated using Chenomx Profiler, which means that the calculated concentrations ideally should equal the added amounts. This makes it possible to evaluate how well the proposed method can remove disturbing background while quantitatively retaining the metabolite signals.

Table S4 Summary of the target signal characteristics of the spiked metabolites

	Chemical class	NMR chemical shift (ppm)	Multiplicity	Interference broad signals/ background	Interference other metabolites
**Asparagine**	amino acid	2.86	dd	no	no
**GABA**	amino acid	1.89	m	no	yes
**Tartaric acid**	organic acid	4.31	s	yes	no
**Threonine**	amino acid	1.31	d	yes	yes
**Xylose**	sugar	5.18	d	yes	no

The metabolites were added in different concentrations, which is mainly because their target signals have different multiplicities and consist of different numbers of protons (Table S5). For example, the signal of threonine belongs to a doublet containing three protons whilst asparagine's signal is part of a doublet of doublets containing only one proton. This means that the target signal of asparagine is only around 2.5 times more intense than that of threonine, despite the ten times difference in concentration. The concentrations were furthermore chosen so that the target signals of all added metabolites should be over the limit of quantification (10×noise) in each sample.

Table S5 Approximate concentrations (µM) in the samples after spiking with 5 µl of each standard solution

	mult., no of H	Sample A	Sample B	Sample C	Sample D	Sample E
**Asparagine**	dd, 1H	400	200	1600	800	100
**GABA**	m, 2H	50	25	200	400	100
**Tartaric acid**	s, 2H	400	800	50	200	100
**Threonine**	d, 3H	10	80	40	20	160
**Xylose**	d, 0.35 H[a]	3200	800	400	200	1600

[a] The target signal belongs to the α-pyranose anomer, which makes up ca 35 % of the total xylose.

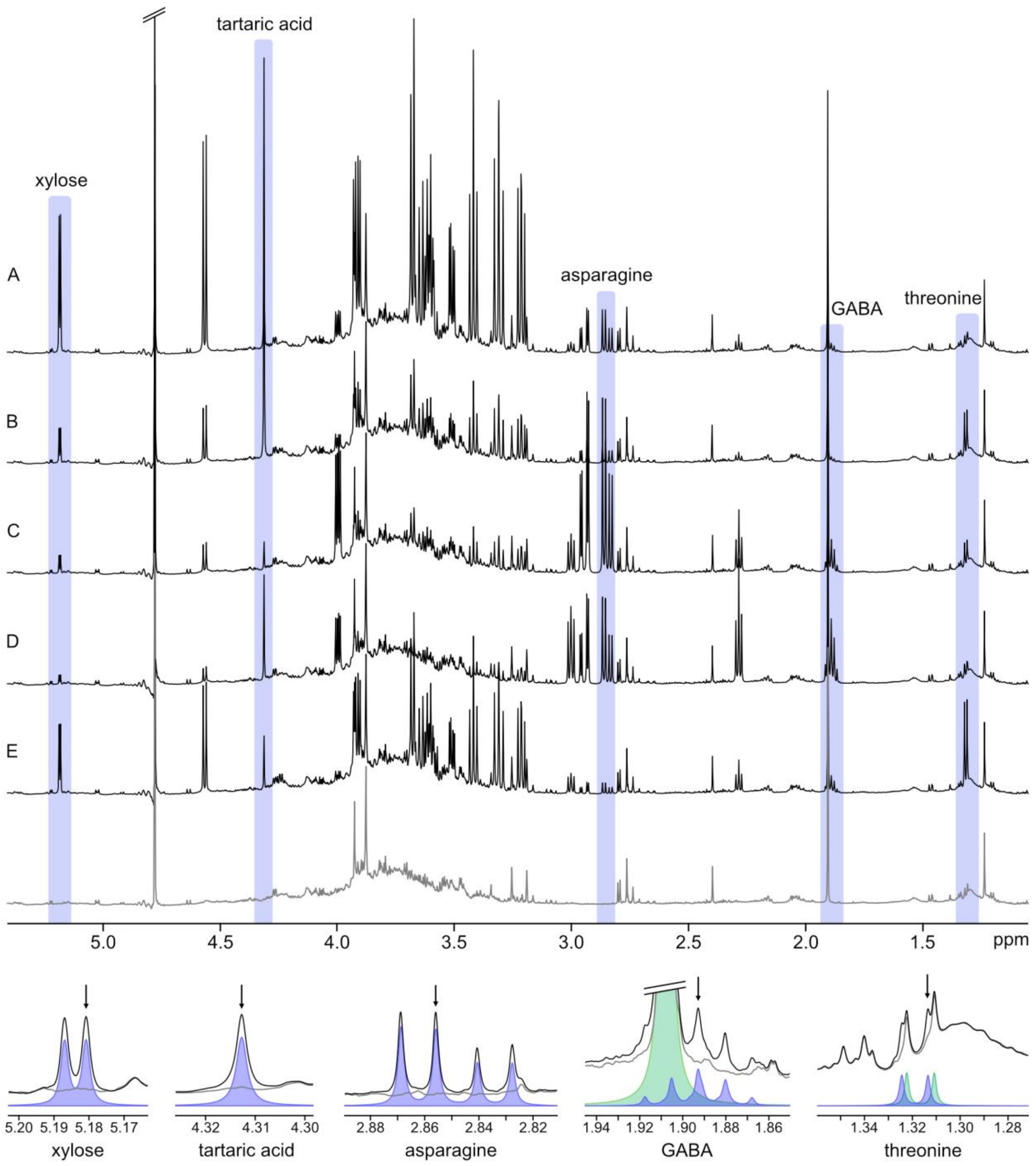

Fig. S11 Upper panel: NMR spectra of the pooled root exudate sample before (grey) and after (black) addition of the spike-in metabolites. Lower panel: Magnification of the target signal regions for the five spiked metabolites. Both the pooled root exudate without addition of spike-in metabolites (grey) and the spiked root exudate with the smallest amount of the respective metabolite added (black) are shown. The corresponding library signals are shown in blue. The target signals used for quantification are marked with arrows. The signals for GABA and threonine overlap with other metabolites (acetic acid and lactic acid, respectively); these are shown in green.

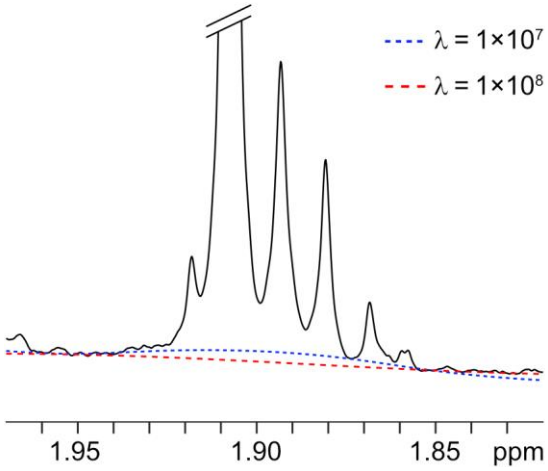

Fig. S12 The effect of two different λ values (1×10^7 and 1×10^8) on the baseline correction around the GABA signal used in AQuA

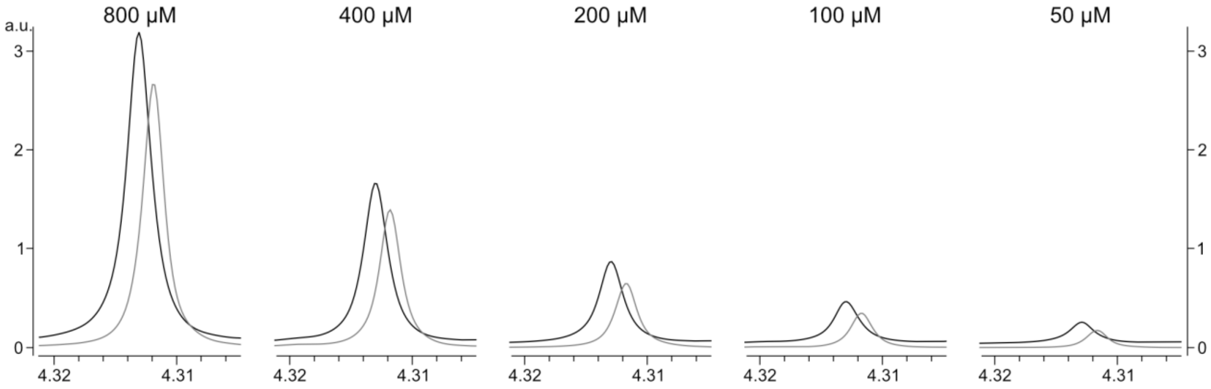

Fig. S13 The signal of tartaric acid in the spectra of the spiked root exudate samples (black) overlaid with the spectra from the corresponding spiked blank samples (grey). The intensities of all spectra have been normalised to the height of the internal standard signal. The pH differed slightly between the root exudates and the blank samples, hence the difference in chemical shift of the tartaric acid signal between the two sample sets.

4.2 Comparison of AQuA results with the actual concentrations

The calculated results from both the spiked root exudate samples and the spiked blank samples (Table S6) were compared with the actual concentrations (Table S7 and S8). Theoretically, the calculated concentrations should equal the actual concentrations for both sample sets, i.e. the slopes should be close to one. Because none of the metabolites was present in the sample to begin with, the intercepts should all be close to zero. In general, the calculated concentrations in the spiked root exudate samples agree better with the calculated concentrations in the corresponding blank samples (Table 1) than with the actual concentrations (Table S7). The difference is smaller for the spiked blank samples; the slopes are generally better when the results are compared with those for the spiked root exudates (Table 1), whereas the R^2 and intercept are comparable between Table 1 and Table S8.

Table S6 Calculated concentrations from the spike-in experiment

		Concentration (μM)			
	**Sample**	**Actual**	**Spiked sample**[a]	**Spiked blank**[b]	**% difference blank-sample**[c]
**Asparagine**	A	401	507	522	2.8
	B	1604	2057	2110	2.5
	C	200	251	257	2.4
	D	802	1021	1042	2.1
	E	100	128	133	3.8
**GABA**	A	50	51	55	8.0
	B	202	206	209	1.7
	C	25	25	27	6.4
	D	403	415	418	0.7
	E	101	104	107	3.3
**Tartaric acid**	A	400	504	438	15.2
	B	50	68	55	22.7
	C	800	988	845	16.9
	D	200	259	206	25.9
	E	100	131	110	18.7
**Threonine**	A	10	15	13	15.4
	B	41	56	51	9.0
	C	82	102	99	3.2
	D	20	27	26	2.2
	E	164	200	198	1.4
**Xylose**	A	3224	3048	3046	0.1
	B	403	398	398	0.1
	C	806	775	774	0.1
	D	202	207	197	5.2
	E	1612	1539	1535	0.2

[a] Concentrations for spiked root exudate samples calculated using an airPLS-extended AQuA including all metabolites listed in Table S1, plus tartaric acid and xylose. Used airPLS parameters: $\lambda_{default}=10^7$, $\lambda_{Thr}=10^5$-10^6, $\lambda_{GABA}=10^8$.

[b] Concentrations for spiked blank samples calculated using an AQuA including only the five spiked metabolites and lactic acid.

[c] Calculated as $100\times|C_{blank}-C_{sample}|/C_{blank}$

Table S7 Comparison of the actual spiked concentrations with the AQuA concentrations obtained for the spiked root exudate samples[a]

Metabolite	Max conc.[b]	R^2	Slope	Intercept	Rel. intercept[c]
**Asparagine**	1604	1.0000	1.28	-5.71	-0.356 %
**GABA**	403	1.0000	1.03	-0.868	-0.215 %
**Tartaric acid**	800	0.9999	1.22	10.2	1.28 %
**Threonine**	164	0.9994	1.20	3.65	2.23 %
**Xylose**	3224	1.0000	0.940	18.9	0.586 %

[a] Results from linear regression. Predictor (x-axis): Actual spiked concentrations. Response (y-axis): Concentrations for spiked root exudate samples calculated using an airPLS-extended AQuA including all metabolites listed in Table S1, plus tartaric acid and xylose. Used airPLS parameters: $\lambda_{default}=10^7$, $\lambda_{Thr}=10^5$-10^6, $\lambda_{GABA}=10^8$.

[b] Actual value (µM) for the spiked sample with the highest concentration

[c] Intercept as percent of the maximum concentration for the metabolite

Table S8 Comparison of the actual spiked concentrations with the AQuA concentrations obtained for the spiked blank samples[a]

Metabolite	Max conc.[b]	R^2	Slope	Intercept	Rel. intercept[c]
**Asparagine**	1604	1.0000	1.32	-5.35	-0.334 %
**GABA**	403	1.0000	1.03	2.36	0.586 %
**Tartaric acid**	800	0.9994	1.06	3.23	0.404 %
**Threonine**	164	0.9999	1.20	1.41	0.864 %
**Xylose**	3224	1.0000	0.941	14.4	0.447 %

[a] Results from linear regression. Predictor (x-axis): Actual spiked concentrations. Response (y-axis): Concentrations for spiked blank samples calculated using an AQuA including only the five spiked metabolites and lactic acid.

[b] Actual value (µM) for the spiked sample with the highest concentration

[c] Intercept as percent of the maximum concentration for the metabolite

One possible reason for the discrepancy between the calculated and actual concentrations is that minor errors in weighing, dilution, and pipetting will lead to the added concentrations differing slightly from the expected amounts. Another explanation is that the accuracy of the AQuA results depends on the level of agreement between the experimental data and the used database, in this case the Chenomx reference library. Before AQuA can calculate metabolite concentrations based on signal heights, it derives metabolite-specific calibration factors from a database. Any discrepancies between the experimental data and the database will be incorporated into the calibration factors and lead to consistent over- or underestimation of the concentration of affected metabolites. This effect is most easily observed in the spectra of the spiked blank samples. These spectra were quantified with AQuA directly, without any additional baseline correction, which means that slopes deviating from one in Table S8 are a result of either database discrepancies or experimental errors, and not necessarily poor algorithm performance. For asparagine and threonine, the calculated concentrations were

significantly higher than the actual concentrations (Table S8). For neither of these compounds was it possible to achieve a good fit for all signals; if the signals belonging to the alpha protons had been chosen to be used in AQuA, the calculated concentrations would have been lower. One explanation for the discrepancy is that the samples in this study were prepared in D_2O whereas the Chenomx spectral library is optimised for samples containing 90 % H_2O (Fig. S13). In general, the signal line widths in the reference library are wider than in the experimental spectra despite careful calibration of the DSS signal line width. Since AQuA calculates metabolite concentrations only based on signal heights, differences in line width can negatively affect accurate quantification.

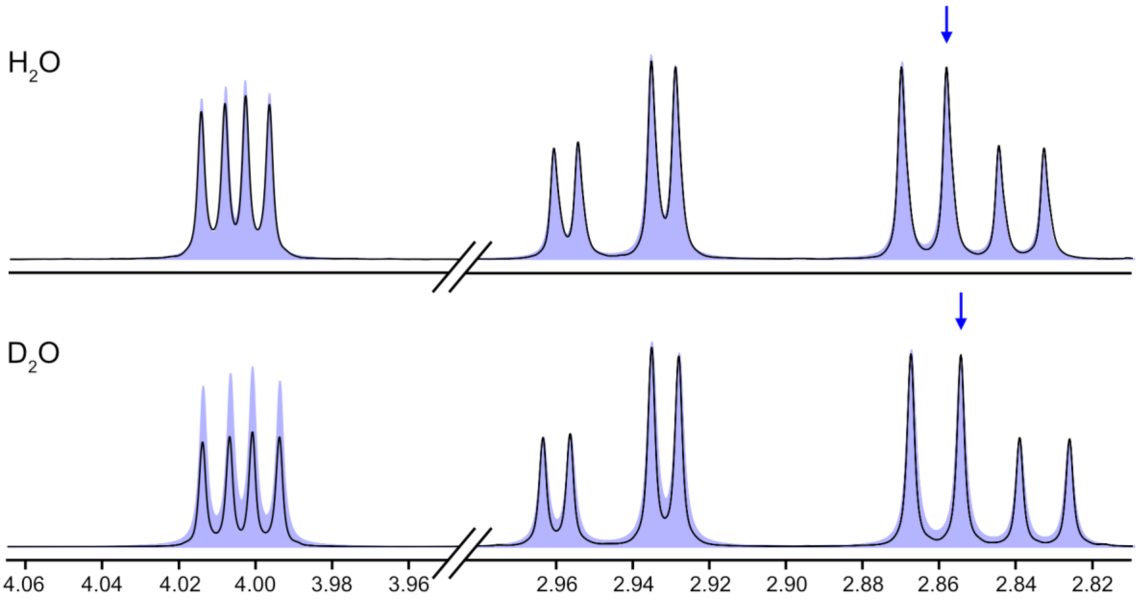

Fig. S14 Experimental spectrum of asparagine (black), dissolved in either 90% H_2O/10% D_2O or 100% D_2O, overlaid with the Chenomx library spectrum (blue) adjusted to the height of the signal that was used in AQuA (marked with arrow). The spectrum recorded in 90 % H_2O displays a better fit with the library spectrum for the alpha proton signal at 4 ppm.

References

Carr, H.Y. & Purcell, E.M. (1954). Effects of diffusion on free precession in nuclear magnetic resonance experiments. *Physical Review*, 94 (3), 630–638. https://doi.org/10.1103/PhysRev.94.630

de Graaf, R.A., Prinsen, H., Giannini, C., Caprio, S. & Herzog, R.I. (2015). Quantification of ^1H NMR spectra from human plasma. *Metabolomics*, 11 (6), 1702–1707. https://doi.org/10.1007/s11306-015-0828-1

Meiboom, S. & Gill, D. (1958). Modified spin-echo method for measuring nuclear relaxation times. *Review of Scientific Instruments*, 29 (8), 688–691. https://doi.org/10.1063/1.1716296
